# Stillbirth outcome capture and classification in population-based surveys: EN-INDEPTH study

**DOI:** 10.1186/s12963-020-00239-8

**Published:** 2021-02-08

**Authors:** Hannah Blencowe, Matteo Bottecchia, Doris Kwesiga, Joseph Akuze, M. Moinuddin Haider, Edward Galiwango, Francis Dzabeng, Ane B. Fisker, Yeetey Akpe Kwesi Enuameh, Bisrat Misganaw Geremew, Tryphena Nareeba, Susannah Woodd, Alexandra Beedle, Kimberly Peven, Simon Cousens, Peter Waiswa, Joy E. Lawn, the Every Newborn-INDEPTH Study Collaborative Group, Peter Byass, Stephen M. Tollman, Hagos Godefay, Joy Lawn, Peter Waiswa, Hannah Blencowe, Judith Yargawa, Joseph Akuze, Ane B. Fisker, Justiniano S. D. Martins, Amabelia Rodrigues, Sanne M. Thysen, Gashaw Andargie Biks, Solomon Mokonnen Abebe, Tadesse Awoke Ayele, Telake Azale Bisetegn, Tadess Guadu Delele, Kassahun Alemu Gelaye, Bisrat Misganaw Geremew, Lemma Derseh Gezie, Tesfahun Melese, Mezgebu Yitayal Mengistu, Adane Kebede Tesega, Temesgen Azemeraw Yitayew, Simon Kasasa, Edward Galiwango, Collins Gyezaho, Judith Kaija, Dan Kajungu, Tryphena Nareeba, Davis Natukwatsa, Valerie Tusubira, Yeetey A. K. Enuameh, Kwaku P. Asante, Francis Dzabeng, Seeba Amenga Etego, Grace Manu, Alexander A. Manu, Obed Ernest Nettey, Sam K. Newton, Seth Owusu-Agyei, Charlotte Tawiah, Charles Zandoh, Nurul Alam, Nafisa Delwar, M. Moinuddin Haider, Md. Ali Imam, Kaiser Mahmud, Angela Baschieri, Simon Cousens, Vladimir S. Gordeev, Victoria Ponce Hardy, Doris Kwesiga, Kazuyo Machiyama

**Affiliations:** 1 Maternal, Adolescent, Reproductive & Child Health (MARCH) Centre, London School of Hygiene & Tropical Medicine, London, UK; 2 Department of Health Policy, Planning and Management, Makerere University School of Public Health, Kampala, Uganda; 3 Centre of Excellence for Maternal Newborn and Child Health Research, Makerere University, Kampala, Uganda; 4 International Maternal & Child Health, Dept. of Women and Children’s Health, Uppsala University, Uppsala, Sweden; 5 Health Systems and Population Studies Division, icddr,b, Dhaka, Bangladesh; 6 IgangaMayuge Health and Demographic Surveillance System, Makerere University Centre for Health and Population Research, Makerere, Uganda; 7 Kintampo Health Research Centre, Kintampo, Ghana; 8 Bandim Health Project, Bissau, Guinea-Bissau; 9 Research Centre for Vitamins and Vaccines, Statens Serum Institut, Copenhagen, Denmark; 10 Department of Clinical Research, Open Patient data Explorative Network (OPEN), University of Southern Denmark, Odense, Denmark; 11 Department of Epidemiology and Biostatistics, Kwame Nkrumah University of Science and Technology, Kumasi, Ghana; 12 Department of Epidemiology and Biostatistics, Institute of Public Health, University of Gondar, Gondar, Ethiopia; 13 Centre of Excellence for Maternal Newborn and Child Health Research, Makerere University, Kampala, Uganda; 14 Department of Public Health Sciences, Karolinska Institutet, Stockholm, Sweden

**Keywords:** Stillbirth, Survey, Measurement, Classification

## Abstract

**Background:**

Household surveys remain important sources of stillbirth data, but omission and misclassification are common. Classifying adverse pregnancy outcomes as stillbirths requires accurate reporting of vital status at birth and gestational age or birthweight for every pregnancy. Further categorisation, e.g. by sex, or timing (intrapartum/antepartum) improves data to understand and prevent stillbirth.

**Methods:**

We undertook a cross-sectional population-based survey of women of reproductive age in five health and demographic surveillance system sites in Bangladesh, Ethiopia, Ghana, Guinea-Bissau and Uganda (2017–2018). All women answered a full birth history with pregnancy loss questions (FBH+) or a full pregnancy history (FPH). A sub-sample across both groups were asked additional stillbirth questions. Questions were evaluated using descriptive measures. Using an interpretative paradigm and phenomenology methodology, focus group discussions with women exploring barriers to reporting birthweight for stillbirths were conducted. Thematic analysis was guided by an a priori codebook.

**Results:**

Overall 69,176 women reported 98,483 livebirths (FBH+) and 102,873 pregnancies (FPH). Additional questions were asked for 1453 stillbirths, 1528 neonatal deaths and 12,620 surviving children born in the 5 years prior to the survey. Completeness was high (> 99%) for existing FBH+/FPH questions on signs of life at birth and gestational age (months). Discordant responses in signs of life at birth between different questions were common; nearly one-quarter classified as stillbirths on FBH+/FPH were reported born alive on additional questions. Availability of information on gestational age (weeks) (58.1%) and birthweight (13.2%) was low amongst stillbirths, and heaping was common. Most women (93.9%) were able to report the sex of their stillborn baby. Response completeness for stillbirth timing (18.3–95.1%) and estimated proportion intrapartum (15.6–90.0%) varied by question and site. Congenital malformations were reported in 3.1% stillbirths. Perceived value in weighing a stillborn baby varied and barriers to weighing at birth a nd knowing birthweight were common.

**Conclusions:**

Improving stillbirth data in surveys will require investment in improving the measurement of vital status, gestational age and birthweight by healthcare providers, communication of these with women, and overcoming reporting barriers. Given the large burden and effect on families, improved data must be made available to end preventable stillbirths.

**Supplementary Information:**

**Supplementary information** accompanies this paper at 10.1186/s12963-020-00239-8.

## Key findings

**Table:** 

**WHAT IS NEW?**
• **What was known already:** Household surveys remain an important source of population-based data on stillbirth in low and middle income countries, but data quality challenges, including omission and misclassification of events, remain. Few studies have examined survey performance of relevant parameters necessary for accurate stillbirth data, notably vital status, gestational age, birthweight and timing of stillbirth. • **What was done:** We undertook a population-based survey of 69,176 women of reproductive age in five countries. We used standard Demographic and Health Survey (DHS) questions and added new questions to identify and categorise stillbirths. Focus group discussions with survey respondents explored barriers/enablers to reporting birthweight for stillbirths in surveys using an interpretative paradigm and phenomenology methodology.
**What was found in the quantitative data?**
• **Existing DHS maternity history questions:** Completeness was high (> 99%) for standard DHS questions used to calculate stillbirth rates: signs of life at birth, gestational age in months and date of the event. • **Additional questions to improve classification of an adverse pregnancy event as a stillbirth:** ○ *Signs of life at birth (misclassification between stillbirths and neonatal deaths):* There was a high proportion of discordant responses between the standard DHS maternity history and additional questions. Discordant responses were more likely for babies classified as stillbirths (around 25%) compared with neonatal deaths (around 3%). ○ *Gestational age (misclassification between miscarriages and stillbirths):* Completeness of reporting of gestational age in weeks for stillbirths (58.1%) was lower than for livebirths (75.4%) and varied by site. ○ *Birthweight* (*misclassification between miscarriages and stillbirths):* Only 13.2% of stillborn babies were reported as weighed at birth. Heaping on multiples of 500 g was common (60.2%) and was more marked for recalled than health card-recorded birthweights.
• **Additional questions to improve categorisation of stillbirths:**
○ *Sex of stillbirth:* Most women (93.9%) were able to report the sex of their stillborn baby. ○ *Timing of stillbirth (antepartum versus intrapartum) or congenital malformations:* Most women could report on fetal movements (92.1%) and skin condition at birth (81.2%) but only 34.6% on the presence of a heartbeat during labour. At an individual level, agreement in classification of intrapartum stillbirth status by question type was very low. The estimated proportion of stillbirths that were intrapartum varied by question and site. Overall 3.1% of stillbirths were reported to have a congenital malformation but women may have found this question difficult to answer—38.3% of women did not respond or said they did not know.
**What was found in the qualitative data?**
• **Perceived value of birthweight:** The perceived value to women of weighing a stillborn baby varied. The majority of women reported no perceived value, but a minority thought that it may help understand why the baby died and prevent recurrence. • **Barriers to reporting birthweight:** *○* Healthcare workers were perceived to ignore, and hence not weigh, stillborn babies. Women who did not perceive the value of weighing stillborn babies may be unlikely to request a birthweight, or retain this information. ○ When weighed, birthweight was not always communicated to the bereaved women, especially if the mother was also sick.
**What next in measurement and research?**
• **Measurement improvement now:** *○* Adding questions on sex of stillborn babies and birthweight should be considered in household surveys. *○* Increasing birthweight measurement of stillborn babies and better communication to women, e.g. verbally and through health cards should be feasible, especially for facility births, and would increase availability of birthweight data for stillbirths in surveys but may require addressing existing stigma and perception around stillbirth. • **Research needed:** In order to improve data which can be used to better inform our understanding and prevention of stillbirth, research is needed firstly to improve the measurement by care providers of vital signs and gestational age to correctly identify stillbirth and reduce misclassification, and vital status in labour to distinguish between antepartum and intrapartum stillbirths. Barriers to the communication of this information to women and families and to survey reporting need then to be understood and addressed.

## Background

More than two and a half million third trimester stillbirths were estimated to occur worldwide in 2015, half during labour (intrapartum) [1, 2]. Yet most stillbirths are preventable. For families and from a public health perspective, preventing these deaths is important, but targeting actions and driving investment requires more data [2–6]. Until recently, stillbirths were not routinely reported or tracked and have received less global attention than neonatal or child deaths. Whilst the target for neonatal mortality reduction was included in the Sustainable Development Goals (SDGs), the stillbirth target was not. This situation is changing. Stillbirths are included in the Every Newborn Action Plan (ENAP), with 194 countries committing at the World Health Assembly in 2014 to reduce stillbirth to 12 per 1000 births by 2030 [7]. Stillbirth rate is a core indicator in the monitoring for the Global Strategy for Women’s, Children’s and Adolescents’ Health and The United Nations (UN) has committed to produce regular national and global stillbirth rate estimates alongside estimates of child mortality [8].

Stillbirths are defined by the International Classification of Disease (ICD) as a baby born with no signs of life with a birthweight ≥ 500 g or at ≥ 22 weeks of gestation [9]. The World Health Organization (WHO) recommends that all stillbirths are counted but late-gestation stillbirths at ≥ 1000 g or ≥ 28 weeks only are included in international comparisons. ICD-10 used birthweight-based criteria in preference over gestational-age; however, these two are not equivalent and in the last two sets of global estimates, gestational age has been used where possible [1]. Accurate stillbirth rates require that every birth event is counted, vital status at birth is known and gestational age or birthweight are available to accurately identify and classify every stillbirth.

Data on stillbirths from low- and middle-income countries (LMICs) rely predominantly on household surveys, notably Demographic and Health Surveys (DHS), which are the only large survey platforms to have systemically collected such data. However, concerns have been raised regarding the quality of stillbirth data within surveys [2, 6, 10] and omission of stillbirths and early neonatal deaths is reported to be common [11, 12]. For more than three decades, DHS used a reproductive calendar to capture pregnancy outcomes; then, in the 7th phase of DHS (DHS-7), additional questions were asked on non-livebirths in the last 5 years after obtaining the woman’s full live birth history (FBH+) [13]. From DHS-8, full lifetime pregnancy history (FPH) is being used in order to better capture stillbirths [10, 13].

Reporting vital status at birth requires distinguishing between very early neonatal deaths and stillbirths, which can be challenging, even for healthcare providers [14, 15], and women may be even less likely to know or have reasons for misreporting it [16]. Misclassification between stillbirths and very early neonatal deaths is therefore a common challenge [11, 17, 18].

**Fig 1:**
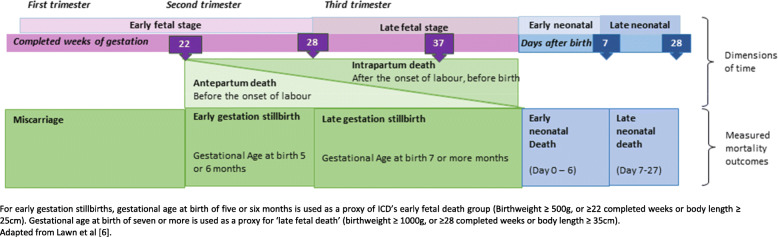
Classification of stillbirths and neonatal deaths in EN-INDEPTH study

Information on gestational age of stillbirths is captured in DHS, but birthweight is only asked for livebirths [19]. Since most surveys collect gestational age in months, not weeks, a threshold of seven or more months is used as a proxy for ≥ 28 weeks to define ‘late gestation stillbirths’. Overall, quality of survey reported gestational age is considered to be low [20–22]. Inaccurate assessment of gestational age may result in misclassification between early and late gestation stillbirths and earlier pregnancy losses or miscarriages (Fig. 1). Despite this, little previous research has assessed gestational age data quality for stillbirths or the feasibility of asking for gestational age in weeks. To our knowledge, no previous studies have examined the feasibility of capturing information on birthweight for stillbirths in surveys.

In addition, to accurately classify an adverse pregnancy outcome as a stillbirth, further information on underlying causes is required to guide programmatic action to end preventable stillbirths [23–25]. In the absence of medical certification of the cause of death in LMICs, verbal autopsy has been the most common method used to collect such information, including for stillbirths [18, 25, 26]. Despite the limitations, verbal autopsy can provide useful information to drive action. For example, knowing that a high proportion of all stillbirths are intrapartum could provide further evidence to support investment in improving access to high-quality intrapartum monitoring and emergency care. So far, very few national household surveys have conducted verbal autopsies of stillbirths [27], and the asking of a limited number of questions regarding the timing of fetal death (antepartum or intrapartum) and whether or not congenital malformations in a standard women’s questionnaire has not yet been assessed.

Disaggregating information by sex is needed to track and close gender gaps; however, no information on the sex of stillborn babies is currently collected in DHS.

This paper is one of a series from the ‘Every Newborn-International Network for the Demographic Evaluation of Populations and their Health’ (EN-INDEPTH) study in five health and demographic surveillance system (HDSS) sites in Africa and Asia. This paper addresses three objectives:

1. ***Existing DHS maternity history questions:*** To describe the use of existing DHS questions for the capture of stillbirths and calculation of stillbirth rates (FBH+/FPH).
2. ***Additional questions:*** To evaluate new questions to improve classification and categorisation of stillbirths including signs of life at birth, gestational age and birthweight, timing of stillbirth (ante- and intra-partum), presence of congenital malformations and sex of the baby.
3. ***Community perceptions:*** To undertake qualitative research on barriers and enablers to reporting birthweight for stillbirths, and how these affect measurement in population-based surveys.

## Methods

### Overall study design and setting

The EN-INDEPTH study involved a cross-sectional, multi-site survey conducted between July 2017 and August 2018, including women aged 15–49 years, and focus group discussions (FGDs) with women and interviewers in five HDSS sites: Bandim in Guinea-Bissau, Dabat in Ethiopia, IgangaMayuge in Uganda, Matlab in Bangladesh and Kintampo in Ghana (Additional file 1.1). The study protocol and results of the primary objective to compare two methods of retrospective recording of pregnancy outcomes in surveys (with women randomised to FBH+ and FPH) have been published elsewhere [10, 28].

The EN-INDEPTH study also investigated the performance of existing or modified survey questions to capture additional pregnancy-related information for a sub-sample of survey respondents with a birth since 1st January 2012. The sub-sample included all women with a neonatal death or pregnancy loss at 5 months or more regardless of whether their mother was randomised to receive a FBH+ or a FPH in the first part of the survey (Additional file 1.2).

Survey data from women and interviewers were collected on Android tablets using Survey Solutions data collection and management system [29]. Interviewers were recruited locally and were familiar with the culture and dialect of the study area. Following completion of data collection, data from the five HDSS sites were anonymised by local HDSS scientists, encrypted and then shared [28]. Quantitative data management and analysis were done using Stata version 15·1. Qualitative data were transcribed using a combination of notes and audio recordings and were coded and analysed using NViVo 12 software.

### Methods by objective

#### Objective 1: To describe the use of existing DHS questions for the capture of stillbirths and calculation of stillbirth rates (FBH+/ FPH)

Consistent with methods used in DHS, all women participants provided information on all livebirths in their lifetime and pregnancy losses since 1st January 2012 (standard DHS-7 FBH+ approach), or all pregnancies in their lifetime regardless of the outcome (FPH approach to be used as standard in DHS-8). The questions asked varied slightly between FBH+ and FPH (see Table 1). In both groups, late gestation stillbirths in the last 5 years were defined as non-livebirths with reported pregnancy duration of seven or more months, with a date of birth within 5 years of the date of the survey using these standard DHS questions. The DHS’s century month code (CMC) system was used to identify events occurring in the 5 years prior to the interview [30]. Stillbirth rates for the 5 years prior to the survey were calculated as the number of late gestation stillbirths/(number of late gestation stillbirths and livebirths) × 1000. Completeness of responses was assessed using descriptive statistics.

**Table 1:** Survey questions used to calculate stillbirth rates in DHS and the EN-INDEPTH survey

DHS standard approach	Domain	Question	Potential responses
DHS-7 FBH+	Vital status at birth	Have you ever had a pregnancy that miscarried, was aborted, or ended in a stillbirth?	Yes, No
		Since January 2012, have you had any other pregnancies that did not result in a livebirth?	Yes, No
	Duration of pregnancy/gestational age	How many months pregnant were you when that pregnancy ended?	Numeric integer (Range: 0–11)
	Event in 5 years prior to survey	When did such pregnancy end?	Months: Single select from list or Don’t Know Years: Numeric integer (Range: 1980–2018)
DHS-8 FPH	Vital status at birth	Was the baby born alive, born dead, or lost before full term?	Born alive, born dead, lost before full term
		*If responded ‘born dead’ then asked:* Did that baby cry, move, or breathe when it was born?	Yes, No
	Duration of pregnancy/gestational age	How many months did this pregnancy last?	Numeric integer (Range: 0–11)
	Event in 5 years prior to survey	On what day, month and year did this pregnancy end?	Days: Numeric integer (Range: 1–31) Months: Single select from list or Don’t Know Years: Numeric integer (Range: 1980–2018)

Questions included above for the FBH+ are the exact questions used in the model questionnaire for DHS-7, asked after the full live birth history. Questions included for the FPH are from the Nepal 2016 DHS FPH module, which are similar to the FPH module included in the core woman’s questionnaire in DHS-8

#### Objective 2: To evaluate new questions to improve classification and categorisation of stillbirths

Consistent with the standard DHS approach, pregnancy duration of ≥7 months was used to calculate overall stillbirth rates above. However, in view of uncertainty around gestational age and consistent with ICD’s recommendation to collect information on all stillbirths ≥500 g or ≥ 22 weeks, we included non-livebirths at ≥ 5 months’ gestation in the analyses of stillbirth classification and differentiate between early gestation (reported pregnancy duration 5 or 6 months) and late gestation (reported pregnancy duration ≥ 7 months) stillbirths.

Further additional questions not routinely asked in standard DHS surveys regarding signs of life at birth, gestational age in weeks and birthweight were asked to all women reporting an early or late gestation stillbirth, or a neonatal death (before 28 days of life) in the 5 years prior to the survey (Table 2). Questions on the sex of the baby were only asked for stillbirths at 6 months or more in the FPH group only. Additional questions adapted from the WHO verbal autopsy tool [34] on the presence of congenital malformations and stillbirth timing (antepartum vs intrapartum) were asked for early and late gestation stillbirths. Assessment of timing of a stillbirth was limited to late gestation stillbirths, to enable comparison with existing global estimates [2].

**Table 2:** Survey questions not in DHS but added to EN-INDEPTH study regarding categorisation of stillbirths

Domain	Question	Source of question	Potential responses	Target population
Vital Status at birth	Did this baby cry, move or breath at birth, even a little? *If responds ‘no’ or ‘don’t know’ to first part:* If this baby did not cry, move, or breathe at birth was he/she born dead?	WHO 2012 and 2014 verbal autopsy tool [31, 32]	Yes, no, don’t know Yes, no, don’t know	All babies classified as neonatal deaths, early fetal deaths or stillbirths based on FBH+ or FPH questions
Gestational Age	How many weeks pregnant were you when this baby was born?	Adapted from WHO 2012 Verbal Autopsy tool [31]	From card, From recall, Don’t know Weeks: numeric integer	All babies selected for additional questions
	Was this baby born before expected? *If responds ‘yes’ to first part:* How many weeks was this baby born before the expected date of delivery? *If does not know weeks:* How many months was this baby born before the expected date of delivery?	Adapted from WHO 2007 Verbal Autopsy tool [33]	Yes, no, don’t know Weeks: numeric integer Don’t know Months: numeric integer Don’t know	
Birthweight	When this baby was born was this baby very large, larger than average, average, smaller than average or very small?	DHS-7 standard question to recent livebirths	Very large, larger than average, average, smaller than average, very small or don’t know	
	Was this baby weighed at birth? *If responds ‘yes’ to first part:* How much did this baby weigh?	DHS-7 standard question to recent livebirths	Yes, no, don’t know Kg from card, Kg from recall, don’t know Kg: numeric integer	
Vital Status at start of labour (intrapartum/ antepartum stillbirth)	Did this baby stop moving in the womb before labour pains started?	Adapted from WHO 2014 and 2016 Verbal Autopsy tool [32, 34]	Yes, no, don’t know	All babies classified as late gestation stillbirths on FBH+ or FPH questions confirming that the baby was born dead on additional questions above.
	Did a birth attendant listen for the baby’s heart beat during labour with an electronic device (Doppler) or cone-shaped stethoscope placed on the abdomen? *If responds ‘yes’ to first part:* Was the baby’s heartbeat present?	WHO Making Every Baby Count – audit and review of stillbirths and neonatal deaths guide [35]	Yes, Doppler Yes, Stethoscope No Don’t know Yes, no, don’t know	
	Was the baby macerated; that is skin peeling or showing signs of decay?	WHO 2007, 2012 and 2014 verbal autopsy tool [31–33]	Yes, no, don’t know	
Presence of congenital malformations	Did this baby have any major malformation at birth? *If responds ‘yes’ to first part:* What kind of malformation did the baby have?^a^	WHO 2007 Verbal Autopsy tool [33]	Yes, no, don’t know Swelling/ defect on back Very large head Very small head Defect of lip/palate Intestines protruding Other Don’t know	All babies classified as early or late gestation stillbirths based on FBH+ or FPH questions
Sex of baby^b^	Was the baby a boy or a girl?	Adapted from DHS-7	Boy, Girl, don’t know	All pregnancy losses at 6 or more months in FPH arm only

*Kg* kilogrammes

^a^DHS-7 asked only of livebirths ‘Is NAME a boy or a girl?’

^b^This question was not asked to women reporting a pregnancy loss at 5 months or earlier

Questions were assessed using descriptive measures including response completeness, proportion of ‘don’t know’, internal consistency, distribution/heaping and pragmatic utility of indicators calculated using the responses. For gestational age assessments, both the new question on gestational age in weeks and standard DHS questions on gestational age in months were included. For comparisons of internal consistency for gestational age questions, gestational age in months was converted to weeks by multiplying by 4.33 and results considered consistent if within 2 weeks. Evidence for statistical differences between responses was assessed using Chi-squared tests.

Results are reported in accordance with STROBE Statement checklists for cross-sectional studies (Additional file 2).

#### Objective 3: To undertake qualitative research on barriers and enablers to reporting birthweight for stillbirths, and how these affect measurement in population-based surveys

Focus group discussions (FGDs) were undertaken with survey respondents including specific questions about weighing of stillborn babies (see Additional file 1.3) [36]. Details of the findings on barriers and enablers to the reporting of adverse pregnancy outcomes, and gestational age are published in accompanying papers in this supplement [20, 36]. A further paper details overall perceptions, barriers and enablers to reporting birthweight in household surveys [37]. In this paper, we undertook thematic analysis to identify community perceptions, practices and barriers and enablers to reporting birthweight specifically for stillbirths. We used an iterative process guided by an a priori codebook and added new codes that emerged during analysis [36]. Themes were summarised and grouped to explore how findings contribute to understanding of the measurement of this key variable required to classify stillbirths in population-based surveys.

## Results

**Fig. 2:**
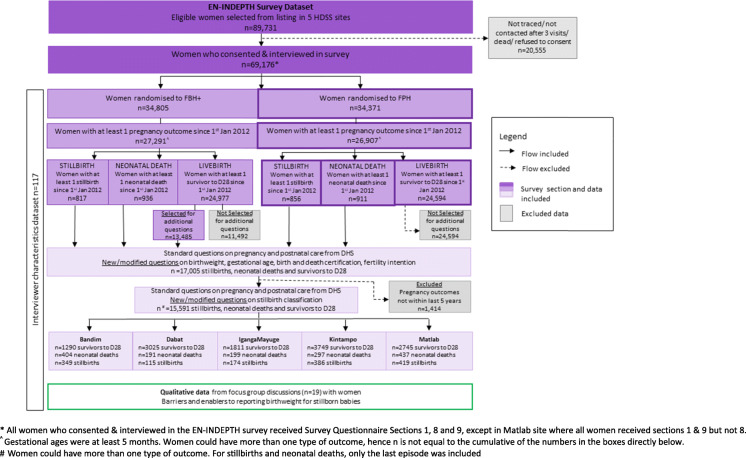
Flow diagram of EN-INDEPTH study population and data included for stillbirth analyses

Information on births was collected for 69,176 women overall, 34,805 using FBH+ and 34,371 using FPH (Fig. 2). Additional data on signs of life at birth, gestational age and birthweight were collected for 410 early gestation stillbirths, 1,033 late gestation stillbirths and 1528 neonatal deaths occurring in the 5 years prior to the survey. Questions on gestational age and birthweight were also asked for 12,620 children surviving the neonatal period. Additional questions on timing of death and congenital malformations were asked for stillbirths.

Interviewer and survey respondents’ socio-economic and demographic characteristics were similar across different outcomes (Additional file 3.1 and 3.2).

### Objective 1: Existing DHS maternity history questions to calculate stillbirth rates (FBH+/ FPH)

Overall 69,176 surveyed women reported 98,483 lifetime livebirths (FBH+ group) and 102,873 lifetime pregnancies resulting in 96,816 livebirths (FPH group). Information on signs of life at birth was available for all FBH+ births, and for all except 181 pregnancies (< 0.2%) in the FPH, which were missing due to system errors. Seven babies (< 0.01%) in the FPH classified as non-livebirths on the ‘Was the baby born alive, born dead, or lost before full term?’ question were reclassified as livebirths based on the further question ‘Did that baby cry, move, or breathe when it was born?’, none of whom were born within the 5 years preceding the survey.

Gestational age in months was available for most non-livebirths apart from 149 pregnancy losses in the FBH+ where the woman reported more than three losses since 1st January 2012 and the survey app was set up to only capture gestational age information for the three most recent losses.

All livebirths in the FBH+ group had a recorded year of birth, 7094 (7.2%) did not have a month of birth and these were imputed (Additional file 3.3). For livebirths in the FPH group, 91 (0.1%) had year and 6809 (7%) had month of birth imputed. All stillbirths recorded since 1st January 2012 had a recorded year of birth in both study arms and 27.1% of stillbirths since 1st January 2012 captured in FBH+ (n = 162) and 33.9% lifetime stillbirths in FPH (n = 596) were missing a month of birth.

Out of 33,528 total births in the 5 years prior to the survey in the FBH+, 508 late gestation stillbirths were reported (SBR = 15.2 per 1000 total births). In the FPH there were 575 late gestation stillbirths out of 33,121 total births (SBR = 17.4 per 1000) [10]. There were large variations across HDSS sites (SBR 8.1 to 20.2 with FBH+ and 10.6 to 25.5 with FPH) [10]. Only a small number of women (*n* = 71) reported more than one late gestation stillbirth in the last five years.

### Objective 2: New questions to improve classification and categorisation of stillbirths

#### Signs of life at birth: misclassification between stillbirths and neonatal deaths

Additional questions regarding presence of signs of life at birth were answered by >99% of women reporting a stillbirth or neonatal death (Additional file 3.4). For the screening question ‘Did THIS BABY cry, move, or breathe at birth, even a little?’, overall, 2% provided a ‘don’t know’ response with evidence of variation by outcome, being more common for early gestation stillbirths (*p* = 0.004). Over 95% of women reporting no signs of life at birth were able to report whether they thought their baby was born dead.

Discordant responses between vital status at birth were more common for babies classified as stillbirths in the FBH+/FPH roster questions (24.6%/23.0%) compared with neonatal deaths (3.4%/2.8%) (overall p < 0.0001) (Table 3). There was weak evidence that FPH babies reported as ‘lost before full term’ were more likely to be classified as livebirths on additional questions compared with babies reported as ‘born dead’ (28.9% compared with 21.0% *p* = 0.03). Most (78.7%) deaths classified as neonatal deaths in the FBH+ or FPH that would be re-classified as stillbirths using the additional questions were day 0 or day 1 neonatal deaths (Table 3 footnote).

**Table 3:** Vital status at birth for neonatal deaths and stillbirths using FBH+/FPH and additional questions

		Vital status assessed using additional questions: ‘Did THIS BABY cry, move, or breathe at birth, even a little?’ if no/unsure ‘was he/she born dead’?			
Vital status assessed using FPH or FBH+	Total	Livebirth	Stillbirth	Don’t know	% discordant answers
**FPH ‘Was the baby born alive, born dead, or lost before full term’**					
‘Born Alive’	762	735	***21***^***a***^	6	2.8%
‘Born Dead’	**552**	**116**	428	8	21.0%
‘Lost before full term’	**183**	**53**	113	16	28.9%
**FBH+ livebirths and pregnancies that ‘miscarried, were aborted, or ended in a stillbirth’ as reported**					
Livebirth	766	731	***26***^***b***^	8	3.4%
Pregnancies reported as ‘miscarried, were aborted, or ended in a stillbirth’	**708**	**174**	509	25	24.6%

Bold text indicates discordant response between FBH+/FPH roster and additional questions. Includes only births in the 5 years preceding the EN-INDEPTH survey

^a^Of the 21 babies reported to have been born alive in the FPH roster (neonatal deaths), but reported as ‘born dead’ with the additional questions (re-classified as stillbirths): 6 were day 0 neonatal deaths, 11 day 1 deaths and 3 were reported to have died on day 2–6, with 1 death on day 8

^b^Of the 26 babies reported to have been born alive in the FBH+ roster (neonatal deaths), but reported as ‘born dead’ with the additional questions (re-classified as stillbirths): 11 were day 0 neonatal deaths, 9 day 1 deaths and 3 were reported to have died on day 2–6, with 1 further death on days 7, 15 and 24 respectively

#### Gestational age and birthweight: misclassification between miscarriages, early and late gestation stillbirths

**Fig. 3:**
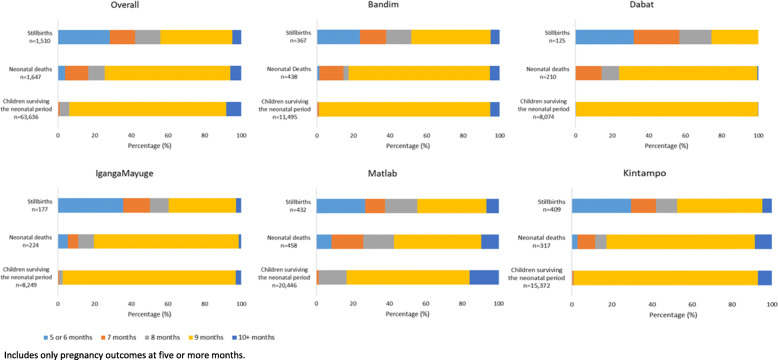
Gestational age in months by outcome, overall and by site (*n* = 66,793)

Gestational age in months was reported for > 99% of pregnancies/births in the last 5 years in the EN-INDEPTH study overall (*n* = 70,973) (Additional file 3.5A and 3.5B). Whilst the gestational age distribution in months showed large between-site variation for children born alive, the distribution was more similar for stillbirths with around 30% of all stillbirths reported as being born at 5 or 6 months of gestation, around 15% at 7 or 8 months, 40% at 9 months and 5% at 10 or more months (Fig. 3).

**Fig. 4:**
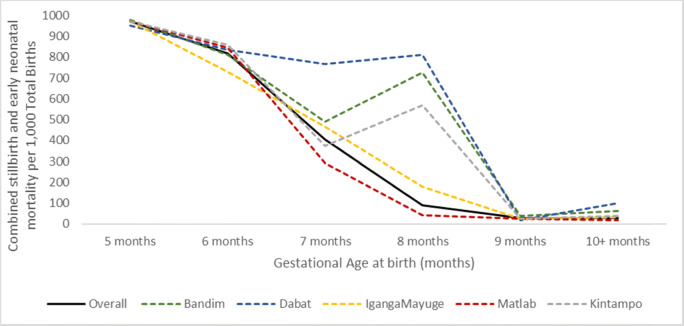
Gestational age–specific combined stillbirth and early neonatal mortality rates, 5 sites (*n* = 66,793)

Overall, gestational age–specific combined stillbirth and early neonatal mortality (perinatal mortality) followed a plausible distribution with very high mortality at early gestations with a minimum at term, and a slight increase in risk for post-term births (Fig. 4). However, in three sites, very few children surviving the neonatal period were reported to have been born at 8 months’ gestation relative to babies who did not survive compared with at 7 or 9 months, resulting in higher gestation-specific mortality for births at 8 months (Additional file 3.6). For the subset asked the additional questions, gestational age in weeks was reported as unknown for 41.6% of stillbirths (*n* = 598) (Additional file 3.7). Amongst those reporting gestational age in weeks at stillbirth, it was exactly four times the reported gestational age in months for 42.5%, and numerically identical to gestational age in months for 2.1%.

Mothers of stillborn babies (early gestation (61.7%), late gestation (83.3%)) and neonatal deaths (94.2%) were less likely to report on perceived size at birth compared with mothers of children surviving the neonatal period (99.6%) (*p* < 0.0001) (Additional file 3.4)*.*

Half (58.1%) of children surviving the neonatal period were reported as weighed, compared with 45.9% of babies who died in the neonatal period and only 13.2% of stillborn babies (*p* < 0.0001) (Table 4). Amongst those reported as weighed, birthweight was not known for one-quarter (25.5%) of babies who died in the neonatal period, 16.5% of children surviving the neonatal period, and 14.3% of stillbirths (*p* < 0.0001). Amongst those reporting a birthweight, a larger proportion of neonatal deaths had a low birthweight (< 2500 g, 28.9%), compared with children surviving the neonatal period (12.7%) or stillbirths (22.2%) (*p* < 0.0001).

**Table 4:** Birthweight reporting for neonatal deaths, stillbirths and children surviving neonatal period (*n* = 15,579)

	Overall number of babies included^a^	Mother reported baby weighed at birth (%)	% of babies weighed with reported birthweight	Overall mean birthweight (kg) (95%CI)	Low birthweight *n* (%)^b^
Children surviving neonatal period	12,618	58.1	83.5	3.08 (3.06–3.10)	775 (12.7)
Neonatal deaths	1527	45.9	74.5	2.92 (2.83–3.01)	151 (28.9)
Late gestation stillbirths	1027	15.9	87.2	3.26 (3.10–3.43)	29 (20.3)
Early gestation stillbirths	407	6.1	76.0	3.04 (2.39–3.68)	7 (36.8)
Overall stillbirths	1434	13.2	85.7	3.24 (3.07–3.40)	36 (22.2)

^a^Data are missing for 2 children surviving neonatal period, 1 neonatal death, 6 late gestation stillbirth and 3 early gestation stillbirth

^b^Percentage of babies with a birthweight whose birthweight is <2500 g

**Fig. 5:**
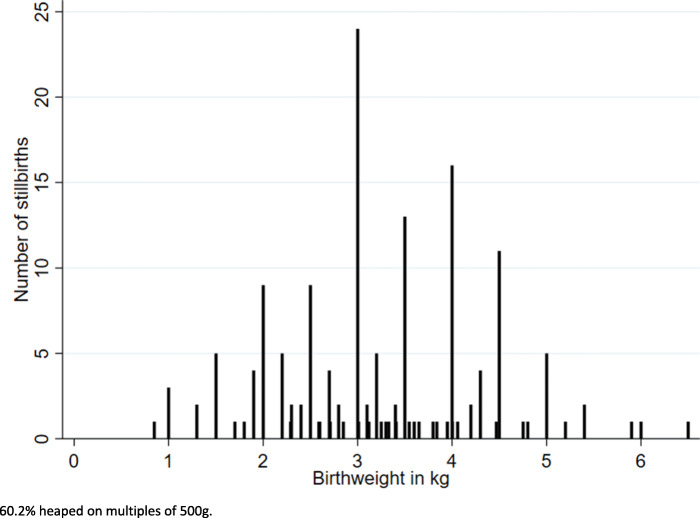
Birthweight heaping for stillbirths in EN-INDEPTH survey (*n* = 161)

There was some evidence that heaping on multiples of 500 g was more marked for stillbirths (60.2% birthweights heaped) compared with 57.9% for neonatal deaths and 53.3% for children surviving the neonatal period (*p* = 0.05) (Fig. 5, Additional files 3.8 and 3.9). Heaping was more marked for recalled birthweights for stillbirths (68.5%) compared with those from a health card (34.4%) (*p* = 0.001) (Additional file 3.10).

#### Timing of stillbirth—antepartum or intrapartum

One-third (33.5%) of women reported fetal movements during labour (suggesting an intrapartum stillbirth), with only 7.9% responding ‘don’t know’ to this question (Table 5). Overall, 43.3% of women with a late stillbirth reported that a birth attendant had listened for a fetal heartbeat during labour (5.7% of homebirths and 55.6% of facility births) (Additional file 3.11). Most women with a late gestation stillbirth (65.4%) did not know if there was a fetal heartbeat during labour; 8.5% reported that fetal heart sounds were heard during labour (Table 5). Two-thirds (67.5%) of women reported no skin maceration, with large variation in ‘don’t know’ responses from 3.5% (Matlab) to 35.8% (Bandim). Excluding ‘don’t know’ responses the estimated proportion of all late gestation stillbirths that were intrapartum varied by question: 36.3% for the fetal movement question, 24.6% for the auscultation question, and 83.1% for the skin appearance question. At an individual level, agreement in classification of intrapartum stillbirth status by question type was very low with fewer than 20% of women reporting consistent answers between the fetal heart rate and other questions, and 35.1% between fetal movements and skin appearance (Additional file 3.12).

**Table 5:** Antepartum and intrapartum stillbirth assessment for late gestation stillbirths in EN-INDEPTH survey (*n* = 846)

Study site	Overall number of stillbirths^a^	Fetal movement questions ***n*** (%)			Fetal auscultation questions^b^ ***n*** (%)			Skin maceration questions^d^ ***n*** (%)			% of late gestation stillbirths that are categorised as intrapartum by question		
		Did not stop moving before start of labour	Stopped moving before start of labour	Don’t know	Fetal heart sounds heard during labour	Auscultated but no fetal heart sounds	Not known if fetal heart sounds present in labour^c^	No maceration	Yes maceration	Don’t know	Fetal Movement questions	Fetal heart sounds questions	Skin appearance questions
Overall	846	33.5	58.6	7.9	8.5	26.1	65.4	67.5	13.7	18.8	36.3	24.6	83.1
Bandim	159	22.0	66.0	11.9	4.4	23.9	71.7	45.3	18.9	35.8	25.0	15.6	70.6
Dabat	71	49.3	43.7	7.0	5.6	12.7	81.7	63.4	7.0	29.6	53.0	30.8	90.0
IgangaMayuge	92	34.8	46.7	18.5	16.3	22.8	60.9	63.0	15.2	21.7	42.7	41.7	80.6
Matlab	287	32.4	62.7	4.9	8.0	22.3	69.7	86.4	10.1	3.5	34.1	26.4	89.5
Kintampo	237	37.1	57.8	5.1	9.7	37.6	52.7	62.4	16.0	21.5	39.1	20.5	79.6

^a^Stillbirth timing questions only asked to subset of women (identified on FBH+/FBH as having a stillbirth and responded that their baby was ‘born dead’ on additional questions. No missing data

^b^Assuming that report of no heartbeat during labour is an antepartum death, and report of heartbeat during labour followed by a birth of a stillborn baby is an intrapartum death. Note limitation that if auscultation occurs only after an intrapartum death, the absence of a fetal heart during labour will be recorded whereas the baby may have been alive at the start of labour

^c^Not reported if fetal auscultation took place or reported auscultation but did not know if fetal heart sounds were heard

^d^Presence of skin maceration is used as a proxy for antepartum stillbirth. Absence of skin maceration (fresh skin appearance) is a proxy of IPSB

#### Presence of congenital malformations

For almost four in 10 stillbirths (38.3%), women did not provide a response or reported they ‘don’t know’ if the baby had a congenital malformation (Additional file 3.13). 45 stillbirths (3.1% of all stillbirths, 5.1% of those with a yes or no response) were reported as having a malformation, with 39 having a single isolated malformation, and six reporting multiple malformations. Over half (53.8%) of women reporting a single isolated malformation did not categorise the malformation into one of the five systems groups asked (Additional file 3.13). Further information on these were only available in three cases, with the responses given: ‘The head was heavily damaged and discoloured’, ‘It seems just like a liver’, ‘damage on the arm’ suggesting that women may have misunderstood what was meant by ‘major malformation’. 20.5% of single malformations (*n* = 8) were reported as spinal defects (neural tube defects) and are potentially preventable.

#### Sex of baby

**Fig. 6:**
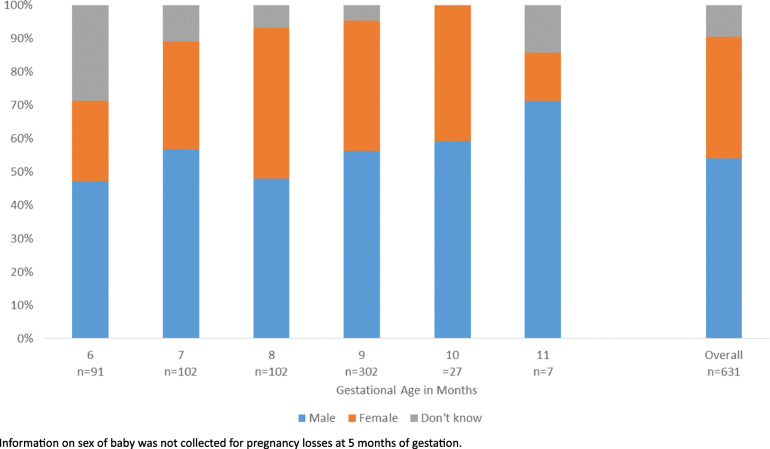
Sex of stillborn babies by gestational age reported in EN-INDEPTH survey (FPH arm only) (*n* = 631)

Most women (93.9%) with a late gestation stillbirth were able to report the sex of their stillborn baby (Fig. 6, Additional file 3.14). The completeness of reporting of fetal sex for stillbirths increased with gestational age from 71.4% at 6 months to 100% at 10 months’ gestation (*p* < 0.0001). Only seven stillbirths were reported at 11 months’ gestation with one of unknown sex.

Overall, 59.6% of stillborn babies with known sex were male, compared with 62.2% of neonatal deaths and 50.0% of children surviving the neonatal period, with similar patterns seen across sites (Additional file 3.14).

### Objective 3: Community perceptions of reporting birthweight for stillbirths

Nineteen focus group discussions were undertaken. Overall, in contrast to the perceived benefits of weighing a liveborn baby [37], there was variation in the perceived value in weighing a stillborn baby reported by women in all sites (Table 6; Additional file 3.15). The majority of women in all sites discussed birthweight in the context of the child only, not considering maternal health, and reported no benefit in weighing for the dead child:

*Since the child is dead and there is nothing else left for the child, it is not necessary to weigh them (Woman, Bandim, Guinea-Bissau)*.

*Not relevant [to take a stillbirth’s weight]. It’s already dead. Why should it be weighed? It just needs to get buried (Woman, IgangaMayuge, Uganda)*.

*It is not necessary [to take a stillbirth’s weight] because it is dead, there is no life in it (Woman, Kintampo, Ghana)*.

**Table 6:** Community perceptions, practices and barriers to reporting birthweight for stillbirths

Theme	Sub-theme	Site(s)	Potential implications for measurement in population-based surveys
Value of measuring birthweight	Lack of perceived value of weighing a stillborn baby	All	This was a very dominant perception, reported by the majority of respondents. Women with such views may be less likely to demand weighing of their babies at birth
	Weighing a stillborn baby may help understand why the baby died and prevent recurrence	Kintampo, IgangaMayuge	Expressed in a minority of sites, but these positive deviants may give some insights into why birthweight for stillbirths may be valued by women
Communication	Separation of mother and baby after birth (sick or stillborn babies, sick mothers)	Bandim	Clearly communicating birthweights to mothers, e.g. verbally and recording in health cards would be necessary to overcome this barrier
Perception of stillbirths	Stillborn babies adversely impact on hospital’s reputation	Matlab	Women perceived that healthcare workers neglected stillborn babies as they were concerned that this would impact on the hospital’s reputation, thus not providing care including weighing of the baby

However, a minority of women in two sites, Kintampo and IgangaMayuge, reported that weighing a stillborn baby had value in helping understand the cause of death and prevent recurrence:

*It is important to weigh…[stillbirths] because the cause of death may not be known…..if they find out that the baby’s low weight contributed to the death, that information can be used to educate women about their diet in order to avoid such problems (Woman, Kintampo, Ghana).*

*No, [weighing a stillborn baby will not make us sad] that will even help to prevent the incident from re-occurring (Woman, Kintampo, Ghana).*

*It may be of importance…[if] it was 9 months, it may be less than a kilogram. Health workers can speculate that may be the child was affected by illnesses while still in the womb (Woman, IgangaMayuge, Uganda).*

Reported barriers to weighing at birth and reporting of birthweight for stillbirths were perceived as common for both home and facility births. In Matlab, mothers reported that healthcare workers neglected stillborn babies, ignoring them instead of measuring birthweight. Mothers attributed this to healthcare workers being concerned that stillborn babies might adversely impact on the reputation of the hospital. In Bandim, women reported that babies who were sick or dead were frequently removed immediately from the mother’s presence, with no-one informing them whether the baby had been weighed, or what the weight was.

*After being born, and it was weighed…. the mother does not have time to see her child because… [he died] or the child is taken away to an incubator, or if the mother becomes sick, it is only afterwards that she can see her child, and from there it is not known if he was weighed or not. (Woman, Bandim, Guinea-Bissau).*

## Discussion

Most of the world’s stillbirths occur in LMICs where measurement is dependent on survey data, yet there have been few studies aiming to improve the quantity and quality of stillbirth data in surveys. The EN-INDEPTH survey included 69,176 women of reproductive age in five countries, and in this paper, we have presented analyses regarding the completeness of stillbirth data and additional questions to improve the stillbirth classification and categorisation.

Completeness was high (>99%) for standard DHS questions used to calculate stillbirth rates. However, stillbirth rate estimates from the survey are lower than expected in these populations especially in the FBH+, suggesting that omission of events remains a challenge [2, 10, 13].

There was a high rate of discordant responses to questions on vital status at birth between FBH+/FPH and additional questions, with responses more likely to be discordant for babies initially classified in FBH+/FPH as stillbirths compared with babies initially classified as neonatal deaths. Assuming the more detailed questions on signs of life at birth are more accurate, in this study, around one-quarter of births classified as ‘stillbirths/ pregnancy losses’ in the maternity history were actually neonatal deaths, with a smaller amount of misclassification in the opposite direction. Discordant responses were most common with the FPH ‘lost before term’ question which may be misinterpreted as liveborn preterm babies who die (28.9% reclassified as neonatal deaths). The FPH in DHS-8 replaced this question with ‘did you have a miscarriage or abortion?’ which are commonly used terms and likely to be better understood by women, though they still require women to distinguish between a ‘miscarriage’ and a very preterm livebirth who subsequently died [38]. Whilst misclassification between stillbirths and neonatal deaths is thought to be common in household surveys, few studies have sought to quantify this. An analysis of the Afghanistan 2010 mortality survey found 2.7% of early neonatal deaths from a FPH were re-classified as stillbirths following a verbal autopsy and 6.4% of stillbirths reclassified as early neonatal deaths [39]. A study in Guinea-Bissau found 8.4% of neonatal deaths identified in an HDSS were reported as stillbirths using a FBH, and 16.9% of HDSS stillbirths were reported as neonatal deaths [40]. In Malawi, 20.5% of FBH+ neonatal deaths were re-classified as stillbirths on verbal autopsy; this study did not include events classified as stillbirths on FBH+ [17]. Further research, comparing survey responses with high-quality facility-based data, and qualitative work to understand better barriers and enablers to women’s reporting of vital status at birth and to understand variation by context, is warranted [41, 42].

Gestational age in months was reported for >99% pregnancies/ births. Gestational age–specific risk of perinatal death followed a plausible distribution but in three sites very few surviving children were reported to have been born at 8 months’ gestation relative to non-survivors, resulting in higher gestation-specific mortality for births at 8 months. Anecdotal reports suggest 8 months being an ‘unlucky’ gestational age, and hence, women whose babies survived may have been reluctant to report that they were born at 8 months. Completeness of reporting of gestational age in weeks for stillbirths (58.1%) was lower than for livebirths (75.4%) and varied by site and was lower when interviewers were explicitly instructed that it was acceptable to record a ‘don’t know’ response if the woman was not able to report in weeks. The overall distribution of gestational age in weeks was plausible, but similar to patterns seen for livebirths in the EN-INDEPTH study, heaping patterns suggest that interviewers may calculate GA in weeks simply by multiplying the number of months by 4 [20]. Estimation of gestational age requires access to early ultrasound pregnancy dating or reliable knowledge of last menstrual period (LMP) which can be challenging [43, 44]. However, access to early pregnancy ultrasound is improving with increasingly portable, robust, lower cost machines which is increasing reliability. In the longer term, collecting gestational age in weeks in surveys—for example, as with birthweight and immunisations, using card data when available—should be possible [20, 45, 46].

Birthweight was reported by few mothers of stillborn babies (13.2%) despite around 60% being facility births. This contrasts with livebirths for which home birth was the largest reported barrier to being weighed [37]. It is possible, as suggested by our qualitative findings, that healthcare workers did not weigh these babies and that women did not request their babies to be weighed, or that they were weighed but that mothers were not informed of the birthweight, or did not perceive this information to be important to retain [37]. Previous research found that despite high coverage of weighing at birth of stillborn babies, women were not able to report birthweight on exit interview [47, 48]. Improving documentation for every stillbirth, including birthweight, for example on handheld health cards, would increase information both to inform women’s future clinical care in subsequent pregnancies, and at the time of household surveys. Birthweight heaping was common with 60.2% of stillbirth birthweights heaped on multiples of 500 g and was more marked for recall than cards [37]. The higher mean birthweight for stillbirths compared with livebirths may represent bias due to the low proportion of stillborn babies with reported birthweights, and it is possible that term intrapartum stillbirths with cephalo-pelvic disproportion were more likely to be weighed than preterm stillbirths.

There was variation in the perceived value of weighing a stillborn baby amongst women, with a minority of women reporting the importance of weighing stillborn babies. Further research is needed to understand why some women perceive this as useful and to use these findings to change overall perceptions and improve weighing of stillborn babies. Perceived barriers to knowing birthweight included healthcare workers’ attitudes affecting both weighing of stillborn babies and communication of birthweight to bereaved women. Improving birthweight data for stillbirths in surveys will require a change in attitudes, as well as improvements in accuracy and recording of birthweights and better communication with women. Collection of accurate birthweight information for stillbirths in surveys could assist with understanding underlying causes of stillbirth and could be useful for identifying implausible gestational ages.

Information on baby’s sex is important as male babies have approximately a 10% higher biological risk of stillbirth than females, but in some cultures with male-sex preference, females have a higher social risk of sex-selective fetocide [16, 24, 49]. Although verbal autopsy studies have found near universal reporting of sex of the stillbirth, prior to this study, it was not known if women would be willing and able to report this in a standard women’s questionnaire. We found most women could answer questions regarding sex of their stillborn baby with a plausible sex distribution, supporting inclusion of this important information in standard DHS surveys.

Fetal movements were reported by most women (92.1%) and also skin condition at birth (81.2%) despite the relatively long recall period of up to 5 years, but only 34.6% knew whether the baby’s heartbeat was present during labour. Unsurprisingly, questions on fetal movements had higher response rate as these are recognisable by women, however the accuracy of these responses in categorising stillbirths is not known. The proportion of stillbirths that are intrapartum using the fetal movement questions were variable from 25.0% in Bandim to 53.0% in Dabat, compared with previous estimates of 51% (33.8-81.8%) in sub-Saharan Africa and 59.3% (32.0–84.0%) in South Asia [2]. Further research is needed to assess the validity of women’s report of fetal movements to assess timing of death for stillbirths, and whether this varies by length of recall period. However, improving quality of intrapartum monitoring, including fetal heart assessment, for all women giving birth in facilities is likely to be the most promising way to improve information on stillbirth timing.

Congenital malformations were reported for 3.1% of stillbirths overall. However, there is some evidence that women may have found this question difficult to answer as 38.3% of women did not respond or said they did not know and free text answers suggested some misunderstanding. In addition, as much stigma and shame remains associated with congenital malformations, it is possible that women were reluctant to report on this [16]. Over half (53.8%) of women reporting a single isolated malformation did not categorise the malformation into one of the five systems groups asked about, and it is possible that the questions may not have been clear, or that the women did not see their baby. In absence of further investigations, only external malformations are likely to be reported, and common conditions, notably cardiac malformations, may be missed. Congenital malformations are an increasingly important, and in many cases preventable, contributor to child mortality. Population-level information is limited in many LMICs; therefore, further research regarding inclusion in surveys should be considered [50, 51].

Strengths of this study include the large survey dataset from five LMICs, with consistent questions and analyses, plus multi-site comparable, qualitative data. To our knowledge, this is the first study to undertake qualitative research alongside a quantitative survey to seek to understand the reporting and measurement of birthweight for stillbirths. We did not include specific discussion topics around the reporting of vital status at birth, timing of stillbirth or gestational age for stillbirths in our focus group discussions, and further research is needed to better understand women’s perceptions of these. Since our study was undertaken with women in HDSS sites who were under regular surveillance, their knowledge about stillbirth may differ from women not under surveillance. In view of the variations in quality and completeness of the HDSS data, we were not able to compare the survey reported outcomes with an external ‘gold standard’, and as such, information reported by women may be biased. Consistent with standard DHS we used a 5-year recall period, and thus, recall of events that took place further from the time of the survey may be less well-recalled. We found substantial inter-site heterogeneity which may be due to differences in trainings and translation of the standard study interviewer manual, or to local contextual differences [13, 52].

The EN-INDEPTH study overall has shown that stillbirth data could be improved now through reducing omission of events by using a FPH, and addressing sociocultural beliefs and psychosocial impacts of stillbirth which affect reporting through carefully adapting tools to local contexts and improving interviewers’ probing, building rapport and empathy skills [10, 36]*.* However, if this information on stillbirths is to be accurate, details of vital status at the start of labour and at birth, gestational age and birthweight are required. This paper has highlighted some of the challenges in obtaining such information in surveys which still need to be addressed to accurately identify which reported adverse pregnancy events are stillbirths, reduce misclassification with early neonatal deaths, and to correctly categorise as antepartum or intrapartum. Women will not be able to report such information in surveys unless these have been accurately measured and communicated to them. With around 80% of all babies globally now born in health facilities, investments and innovations to improve the measurement and recording for this information will be an important next step for improving stillbirth data overall across all data systems [48]. Communicating this information to bereaved women and their families in a sensitive manner in both verbal and where possible written-form, ideally both around the time of birth and at follow-up visits as part of a package of supportive bereavement care will be essential to improving stillbirth data in household surveys [53].

## Conclusions

Myths that women cannot or will not report stillbirths should be left behind. Adding questions on sex of the stillborn baby and birthweight should also be considered in household surveys now. Improving birthweight data for stillborn babies, and better communication to women, for example through health cards or data linkage should be feasible. More innovation is needed to improve gestational age measurement for both live and stillborn babies.

More research is needed to improve classification of stillbirth timing and is dependent on higher quality care at birth with routine fetal heart rate assessment and communication of findings to the women. The initial priority for this should be to improve care and data in hospitals, where around 80% of babies are now born. Given the major burden of stillbirths, and effect on families, more investment is needed in both health facility and household survey measurement to increase data quantity and quality and link data to action.

## Supplementary Information

**Additional file 1.** Additional methods. 1.1: Background overview of the five HDSS sites. 1.2: Details of selection of women with a livebirth surviving the neonatal period. 1.3: Details of qualitative methods for FGD in EN-INDEPTH study.

**Additional file 2.** STROBE guidelines checklist.

**Additional file 3.** Additional results. 3.1: Maternal characteristics of the births in the five years preceding the survey for five sites. 3.2: Interviewer characteristics of births in the five years preceding the survey for five sites. 3.3: Distribution of missing year or month of pregnancy outcome by FBH+/FPH groups. 3.3: Distribution of missing year or month of pregnancy outcome by FBH+/FPH groups. 3.5A: Distribution of reported gestational age in months by outcome (n=70,973). 3.5B: Distribution of reported gestational age in months by outcome (gestation ≥5 months) (n=66,793). 3.6: Gestational age specific stillbirth, perinatal and early neonatal mortality rates, overall and site specific. 3.7: Summary of reporting of gestational age in weeks for stillbirths. 3.7A: Distribution of reported gestational age in weeks by outcome (n=15,591). 3.7B: Internal consistency of gestational age reported in weeks and months by site and by outcome. 3.8: Birthweight distribution by outcome. 3.9: Heaping indices for birthweight, by outcome (n=6,749). 3.10: Comparison of heaping on multiples of 500g by card and recall by outcome. 3.11: Reported intrapartum monitoring for late gestation stillbirths, by site (n=846). 3.12: Individual-level consistency between intrapartum stillbirth classification, by site. 3.12: Individual-level consistency between intrapartum stillbirth classification, by site. 3.14: Reporting of child’s sex by site and outcome (FPH group only. n=631). 3.14: Reporting of child’s sex by site and outcome (FPH group only. n=631). 3.15: Barriers and enablers to birthweight measurement in surveys for stillbirths by site.

**Additional file 4.** Ethical approval of local Institutional Review Boards.

## Data Availability

Data sharing and transfer agreements were jointly developed and signed by all collaborating partners. The datasets generated during the current study are deposited online at 10.17037/DATA.00001556 with data access subject to approval by collaborating parties.

## Abbreviations

DHS: Demographic and health surveys
ENAP: Every newborn action plan
EN-INDEPTH: Every Newborn-International Network for the Demographic Evaluation of Populations and their Health
FBH+: Full birth history (+ denotes additional questions on pregnancy losses)
FGDs: Focus group discussions
FPH: Full pregnancy history
HDSS: Health and demographic surveillance system
ICD: International classification of diseases
ID: Identification number
LMICs: Low- and middle-income countries
SDGs: Sustainable development goals
UN: United Nations
VA: Verbal autopsy
WHO: World Health Organization
