# Supplementary material for: Stillbirth outcome capture and classification in population-based surveys: EN-INDEPTH study
Source: Popul Health Metr. 2021 Feb 8;19(Suppl 1):13. doi: 10.1186/s12963-020-00239-8 (PMC7869203; doi:10.1186/s12963-020-00239-8)
Supplement: Supplementary file 1 — Additional file 1. Additional methods. 1.1: Background overview of the five HDSS sites. 1.2: Details of selection of women with a livebirth surviving the neonatal period. 1.3: Details of qualitative methods for FGD in EN-INDEPTH study. [file 12963_2020_239_MOESM1_ESM.docx]

# Additional file 1: Additional methods

## Additional file 1.1: Background overview of the five HDSS sites

|  | **Bandim** | **Dabat** | **IgangaMayuge** | **Matlab** | **Kintampo** |
| --- | --- | --- | --- | --- | --- |
| **Site Information** | | | | | |
| Country | Guinea Bissau | **Ethiopia** | Uganda | Bangladesh | Ghana |
| HDSS start year | 1978 | **1996** | 2004 | 1966 | 1994 |
| Location | Guinea-Bissau, covering rural and urban | Gondor, Amhara regional state, 821 km northwest of Addis Ababa and 75km north | Iganga and Mayuge districts, approximately, 120km east of capital, Kampala along Kenya-Uganda highway | Matlab Upazila, in Chandpur district, 55km southeast of capital, Dhaka | Within the Kintampo North Municipality and the Kintampo South District of the Brong Ahafo (now Bono East) region of Ghana |
| Population | 180,000 | 69,000 | 83,000 | 230,000 | 153,000 |
| Households | 36,000 | 16,000 | 16,000 | 53,000 | 32,000 |
| Total Fertility Rate | 4.3 | 3.8 | 4.3 | 2.6 | 4.1 |
| **Data capture process** | | | | | |
| Frequency of surveillance rounds | Urban: Monthly  Rural: 2/year (more frequent in some regions) | 2/year | 2/year | 6/year | 1/year |
| Frequency of re-census | Urban: Intervals of 2-7 years dependent on funding; Rural: continuous | Every 7 years | Each update round (Twice a year) | 8 years or more | Last census 2003 |
| Methods for pregnancy identification | Each woman was asked about her pregnancy status and a pregnancy ID is given if found pregnant. | Local guides report pregnancies and births within 48 hours after they have been identified. 83% of the informants are female | Has 64 Community based “scouts”  and Village Health Teams. Both male and female scouts are used | The women age 15-49 years old are given a urine test for pregnancy if pregnancy status is unknown. 100% of the enumerators are female | Community key informants are used to notify pregnancies. Each woman is asked about her pregnancy status |
| Facility births | Urban: 65%  Rural: 39% | 17% | 64% | 69% (Intervention area: 87%; Comparison Area: 50%) | 61% |
| Links to facility data | In national hospital, not in rural | Pilot study ongoing | Not currently | Matlab hospital only (17% of births). The hospital is in the intervention area.^1^ | No |
| Data collection | Paper-based | Piloting tablet since mid-2018 | Paper-based | Electronic (Galaxy Tablet) | Paper-based |
| **Availability of outcome indicators** | | | | | |
| Stillbirths | Yes | Yes | Yes | Yes | Yes |
| Neonatal deaths | Yes | Yes | Yes | Yes | Yes |
| Birthweight | Yes - Urban only | Yes | Yes | Yes | Yes – Since 2015 |
| Gestation Age (GA) | Yes – at pregnancy registration | Yes | Yes | Yes | Yes |
| Birth certificate | Yes – On a subset around 400 women | No | No | Yes | No |
| Death certificate | Urban: Yes – asked with verbal autopsy | No | Yes – asked with verbal autopsy | Yes | Yes – asked with verbal autopsy |
| Miscarriages/ Abortions | Yes - Miscarriages only | Yes - Abortions differentiated from miscarriages | Yes - Miscarriages only | Yes - Abortions differentiated from miscarriages | Yes - Abortions differentiated from miscarriages |

^1^ Nurul Alam., et al, Health and Demographic Surveillance System (HDSS) in Matlab, Bangladesh, International Journal of Epidemiology, Volume 46, Issue 3, June 2017, Pages 809–816, <https://doi.org/10.1093/ije/dyx076>

## Additional file 1.2: Details of selection of women with a livebirth surviving the neonatal period

Initial piloting of the EN-INDEPTH survey app tool found it long to administer. In order to reduce the overall time that interviewers spent on interviews, whilst still reaching the target sample size for the main randomised comparison between FBH+ and FPH, only a subset of women who had at least 1 livebirth that survived the neonatal period since 1^st^ January 2012 were selected to receive the pregnancy and birth module in their questionnaire.

In view of the app set up, it was not possible to select women from the FPH arm for this part of the survey; therefore, only women who were in the FBH+ were eligible for selection. Selection was made at random by the app, with the initial intention that 30% of all women interviewed in the FBH+ arm who had a livebirth surviving the neonatal period (i.e. that did not result in a neonatal death) since 1^st^ January 2012 would be selected to receive the pregnancy and birth questionnaire module after the roster.

The sampling of women with an eligible livebirth was performed differently across the sites. In Dabat, all women interviewed in the FBH+ with an eligible livebirth were selected to receive the pregnancy and birth module. In Matlab and Bandim, throughout the whole data collection period, 30% of women in the FBH+ with an eligible livebirth were selected to receive the pregnancy and birth module. In IgangaMayuge and Kintampo, for the first 1 – 2 months of data collection, all women interviewed in the FBH+ with an eligible livebirth were selected to receive the pregnancy and birth module; for the rest of the data collection period, only a random selection of 30% of such women were selected.

As a result of the variation in the sampling across sites, 50% of women in the FBH+ arm with an eligible livebirth received the pregnancy and birth module. The proportion that completed the module is detailed by site, below:

|  | **Number of women in FBH+ arm completing pregnancy and birth module** | **Percentage of eligible women in FBH+ arm completing pregnancy and birth module** | **Percentage contribution to total surviving livebirths by site** |
| --- | --- | --- | --- |
| **Bandim** | 1,316 | 28.3% | 9.8% |
| **Dabat** | 3,357 | 99.4% | 24.9% |
| **IgangaMaguye** | 1,917 | 64.3% | 14.2% |
| **Matlab** | 2,936 | 29.6% | 21.8% |
| **Kintampo** | 3,951 | 66.7% | 29.3% |
| **Total** | 13,477 | 50.2% | 100.0% |

## Additional file 1.3: Details of qualitative methods for FGD in EN-INDEPTH study

To identify community perceptions, practices, and barriers to reporting outcomes, and how these contribute to understanding of the measurement of any outcome in population-based surveys, 28 Focus Group Discussions (FGDs) were conducted with 82 EN-INDEPTH survey interviewers and supervisors and 172 women between February and August 2018. The number of FGDs undertaken was similar across the sites.

The FGDs explored women’s (respondents) and survey interviewers’ experiences with the EN-INDEPTH survey data collection process, as well as attitudes, knowledge, and practices around reporting and disclosure of pregnancy and adverse pregnancy outcomes and associated enablers and challenges.

**Participant selection**

Women were purposively selected from the pool of respondents who had participated in the EN-INDEPTH survey to ensure diversity by age, place of residence (urban/rural), and experience of APOs.

While all sites recruited participants face-to-face, telephone calls were also made as part of recruitment in Matlab and IgangaMayuge and written information was also left in Bandim if the respondent was not present. In Bandim, four out of 24 women approached in the rural areas and 19 out of 30 approached in the urban areas did not come for the FGDs. There were no refusals or drop outs in the other four sites.

**Data collection**

FGD guides were developed by a multi-country qualitative working group. The women’s FGD guide examined experiences with the EN-INDEPTH survey, how they and others disclose pregnancy and APOs, perceptions on gestational age and birth weight and knowledge and practices around pregnancy and APOs. The data collectors guide considered experiences with the survey process and collecting data on pregnancy and APOs (Additional file 1). The DHS interviewer training manual informed the development of a standard FGD training manual, which was used to train moderators and note-takers in all sites except Bandim. FGD guides were translated into the local language.

FGDs were held in accessible places, including within the community (under trees, sheds, courtyards), at nearby health facilities and at the HDSS offices. Efforts to ensure privacy were made, with only respondents and researchers present. FGDs typically lasted between 1.5 – 2 hours and no repeat FGDs were conducted. Notes from FGDs were written and discussions recorded using a tablet or tape recorder, in addition to field notes. Upon completion, the team transcribed the FGDs in the survey language using a combination of notes and audio recordings. These were then translated into English but were not returned to participants for comments.

The following questions regarding birthweight were asked in the women’s FGDs as part of the full EN-INDEPTH FGD interview guide available at: <https://doi.org/10.17037/DATA.00001556> ________________________________________________________________________________

**Woman’s FGD discussion guide**

**Part E: Gestational age and Birth weight**

In some cases when a woman is pregnant, babies are born too early or too small. We would like to learn from you a few things about this topic.

1. In this community do people think that it is important to count gestational age (from the first day of the woman's last menstrual cycle to the current date/ date when last pregnancy ended?)

- **Probe:** Why is it important? /Why is it not important? If it is important, is it easy for women to count this in this community? Why/ Why not?

1. In this community, do people think that weighing babies when they are born is important?

- **Probe:** Why is it important/ why is it not important? If it is important, how does a mother find out her baby’s birthweight in this community? Are there any problems with getting a baby weighed?
- **Probe:** What about if the baby is stillborn? Why is it important / why is it not important?

**Research team and reflexivity**

In all sites, the interview teams were led by researchers with either Master’s or PhD degrees, who jointly formed a collaborative, consistent, multi-country qualitative working group. While the FGDs were organized by the HDSS teams, which are known within the local communities, there was no direct personal relationship between researchers and respondents. All those involved in data collection including moderators and note-takers were nationals of the respective country. In three HDSS sites, work was led by a staff member with experience in conducting qualitative research. HDSS staff in each site internally recruited experienced FGD moderators and note takers with fluency in the most commonly spoken language, apart from Bandim where they were externally recruited. In Bandim alone, data analysis was undertaken by non Bissau-Guinean researchers with support in understanding the local context from the FGDs moderator and the Bandim HDSS team. The lead researchers were not moderators or note takers, although they attended the FGDs except in Bandim. In IgangaMayuge and Dabat, moderators were males, with females in Matlab and Kintampo, while Bandim had both sexes. Note takers were female in Matlab and Bandim, while Kintampo, IgangaMayuge and Dabat had male note takers.

**Data analysis**

This study used an interpretative paradigm which aims to understand the ways in which people behave, what things mean to them and how they interpret the world and phenomenology methodology to seek to understand peoples’ ‘lived’ experiences. A data management and analysis plan was jointly developed by the multi-site qualitative working group and used across all sites. Both inductive and deductive coding were used. Thematic analysis of the English transcripts was conducted in NViVo version 12 using an iterative process guided by an a priori codebook based on the research teams’ experiences and published literature. Additionally, new codes were included for themes identified during analysis.

Coding for each site was done independently by two coders who met regularly face-to-face and online to discuss identified codes and themes. These coders were the lead researchers for the work in each site, except Bandim where analysis was led by DK, the overall lead researcher for this qualitative study and AB who was externally recruited. The multi-site qualitative working group (most of whom were involved in coding) had conference calls to discuss and synthesize findings, culminating in a face-to-face analysis meeting in February 2019. In these discussions, teams were able to compare coding and agree on identification of themes. Data saturation was discussed and noted by each analysis team in the different sites

**References**

1. Haider et al: **Gestational age validity and feasibility in women’s report survey data: EN-INDEPTH study**. *BMC Population Health Metrics* 2020, **In press as part of EN-INDEPTH supplement**.
