# Supplementary material for: Stillbirth outcome capture and classification in population-based surveys: EN-INDEPTH study
Source: Popul Health Metr. 2021 Feb 8;19(Suppl 1):13. doi: 10.1186/s12963-020-00239-8 (PMC7869203; doi:10.1186/s12963-020-00239-8)
Supplement: Supplementary file 3 — Additional file 3. Additional results. 3.1: Maternal characteristics of the births in the five years preceding the survey for five sites. 3.2: Interviewer characteristics of births in the five years preceding the survey for five sites. 3.3: Distribution of missing year or month of pregnancy outcome by FBH+/FPH groups. 3.3: Distribution of missing year or month of pregnancy outcome by FBH+/FPH groups. 3.5A: Distribution of reported gestational age in months by outcome (n=70,973). 3.5B: Distribution of reported gestational age in months by outcome (gestation ≥5 months) (n=66,793). 3.6: Gestational age specific stillbirth, perinatal and early neonatal mortality rates, overall and site specific. 3.7: Summary of reporting of gestational age in weeks for stillbirths. 3.7A: Distribution of reported gestational age in weeks by outcome (n=15,591). 3.7B: Internal consistency of gestational age reported in weeks and months by site and by outcome. 3.8: Birthweight distribution by outcome. 3.9: Heaping indices for birthweight, by outcome (n=6,749). 3.10: Comparison of heaping on multiples of 500g by card and recall by outcome. 3.11: Reported intrapartum monitoring for late gestation stillbirths, by site (n=846). 3.12: Individual-level consistency between intrapartum stillbirth classification, by site. 3.12: Individual-level consistency between intrapartum stillbirth classification, by site. 3.14: Reporting of child’s sex by site and outcome (FPH group only. n=631). 3.14: Reporting of child’s sex by site and outcome (FPH group only. n=631). 3.15: Barriers and enablers to birthweight measurement in surveys for stillbirths by site. [file 12963_2020_239_MOESM3_ESM.docx]

# Additional file 3: Additional results

## Additional file 3.1: Maternal characteristics of the births in the five years preceding the survey for five sites^1^

|  | Overall | | Children surviving the neonatal period | | Neonatal death | | Late gestation stillbirth | | Early gestation stillbirth | |
| --- | --- | --- | --- | --- | --- | --- | --- | --- | --- | --- |
| ***Site*** | n | % | n | % | n | % | n | % | n | % |
| Bandim | 2043 | 13.1 | 1,290 | 10.2 | 404 | 26.4 | 267 | 25.9 | 82 | 20.0 |
| Dabat | 3331 | 21.4 | 3,025 | 24.0 | 191 | 12.5 | 76 | 7.4 | 39 | 9.5 |
| IgangaMayuge | 2184 | 14.0 | 1,811 | 14.4 | 199 | 13.0 | 112 | 10.8 | 62 | 15.1 |
| Matlab | 3601 | 23.1 | 2,745 | 21.8 | 437 | 28.6 | 307 | 29.7 | 112 | 27.3 |
| Kintampo | 4432 | 28.4 | 3,749 | 29.7 | 297 | 19.4 | 271 | 26.2 | 115 | 28.1 |
|  |  |  |  |  |  |  |  |  |  |  |
| ***Woman age*** | n | % | n | % | n | % | n | % | n | % |
| 15-19 | 665 | 4.3 | 542 | 4.3 | 68 | 4.5 | 40 | 3.9 | 15 | 3.7 |
| 20-24 | 3254 | 20.9 | 2625 | 20.8 | 351 | 23.0 | 201 | 19.5 | 77 | 18.8 |
| 25-29 | 3957 | 25.4 | 3213 | 25.5 | 393 | 25.7 | 248 | 24.0 | 103 | 25.1 |
| 30-34 | 3479 | 22.3 | 2782 | 22.0 | 321 | 21.0 | 271 | 26.2 | 105 | 25.6 |
| 35+ | 4235 | 27.2 | 3457 | 27.4 | 395 | 25.9 | 273 | 26.4 | 110 | 26.8 |
| *missing* | 1 | 0.0 | 1 | 0.0 | 0 | 0.0 | 0 | 0.0 | 0 | 0.0 |
|  |  |  |  |  |  |  |  |  |  |  |
| ***Education level*** | n | % | n | % | n | % | n | % | n | % |
| No education | 4818 | 30.9 | 4039 | 32.0 | 385 | 25.2 | 304 | 29.4 | 90 | 22.0 |
| Primary only | 5130 | 32.9 | 4123 | 32.7 | 523 | 34.2 | 332 | 32.1 | 152 | 37.1 |
| Secondary | 4644 | 29.8 | 3646 | 28.9 | 523 | 34.2 | 344 | 33.3 | 131 | 32.0 |
| Higher | 999 | 6.4 | 812 | 6.4 | 97 | 6.4 | 53 | 5.1 | 37 | 9.0 |
|  |  |  |  |  |  |  |  |  |  |  |
| ***Wealth Quintile*** | n | % | n | % | n | % | n | % | n | % |
| Lowest | 3776 | 24.2 | 3080 | 24.4 | 342 | 22.4 | 264 | 25.6 | 90 | 22.0 |
| Quintile 2 | 3196 | 20.5 | 2546 | 20.2 | 336 | 22.0 | 229 | 22.2 | 85 | 20.7 |
| Quintile 3 | 3038 | 19.5 | 2465 | 19.5 | 302 | 19.8 | 191 | 18.5 | 80 | 19.5 |
| Quintile 4 | 2868 | 18.4 | 2324 | 18.4 | 275 | 18.0 | 196 | 19.0 | 73 | 17.8 |
| Highest | 2713 | 17.4 | 2205 | 17.5 | 273 | 17.9 | 153 | 14.8 | 82 | 20.0 |
|  |  |  |  |  |  |  |  |  |  |  |
| ***Parity*** | n | % | n | % | n | % | n | % | n | % |
| Para 1^2^ | 3038 | 19.5 | 2654 | 21.0 | 168 | 11.0 | 82 | 7.9 | 134 | 32.7 |
| Para 2 | 3474 | 22.3 | 2732 | 21.7 | 405 | 26.5 | 244 | 23.6 | 93 | 22.7 |
| Para 3 | 2686 | 17.2 | 2103 | 16.7 | 292 | 19.1 | 220 | 21.3 | 71 | 17.3 |
| Para 4 | 1930 | 12.4 | 1526 | 12.1 | 211 | 13.8 | 158 | 15.3 | 35 | 8.5 |
| Para 5+ | 4463 | 28.6 | 3605 | 28.6 | 452 | 29.6 | 329 | 31.9 | 77 | 18.8 |

^1^ Includes 15,591 birth outcomes for which additional questions were asked in the survey.

^2^ Includes the 23 cases of early gestation stillbirth in a woman with no previous live or stillbirths.

## Additional file 3.2: Interviewer characteristics of births in the five years preceding the survey for five sites

|  | Overall | | Children surviving the neonatal period | | Neonatal death | | Late gestation stillbirth | | Early gestation stillbirth | |
| --- | --- | --- | --- | --- | --- | --- | --- | --- | --- | --- |
| **Gender** | n | % | n | % | n | % | n | % | n | % |
| Male | 5200 | 33.4 | 4287 | 34.0 | 437 | 28.6 | 333 | 32.2 | 143 | 34.9 |
| Female | 10007 | 64.2 | 7998 | 63.4 | 1070 | 70.0 | 683 | 66.1 | 256 | 62.4 |
| *missing* | 384 | 2.5 | 335 | 2.7 | 21 | 1.4 | 17 | 1.7 | 11 | 2.7 |
| **Age** |  |  |  |  |  |  |  |  |  |  |
| <20 | 311 | 2.0 | 243 | 1.9 | 39 | 2.6 | 22 | 2.1 | 7 | 1.7 |
| 20 – 24 | 2593 | 16.6 | 2014 | 16.0 | 315 | 20.6 | 194 | 18.8 | 70 | 17.1 |
| 25 – 29 | 3723 | 23.9 | 3075 | 24.4 | 338 | 22.1 | 205 | 19.9 | 105 | 25.6 |
| 30 – 34 | 3956 | 25.4 | 3274 | 25.9 | 339 | 22.2 | 249 | 24.1 | 94 | 22.9 |
| 35+ | 4624 | 29.7 | 3679 | 29.2 | 476 | 31.2 | 346 | 33.5 | 123 | 30.0 |
| *missing* | 384 | 2.5 | 335 | 2.7 | 21 | 1.4 | 17 | 1.7 | 11 | 2.7 |
| **Education level** |  |  |  |  |  |  |  |  |  |  |
| Primary | 594 | 3.8 | 548 | 4.3 | 30 | 2.0 | 12 | 1.2 | 4 | 1.0 |
| Secondary | 9670 | 62.0 | 7850 | 62.2 | 917 | 60.0 | 647 | 62.6 | 256 | 62.4 |
| Higher | 4943 | 31.7 | 3887 | 30.8 | 560 | 36.7 | 357 | 34.6 | 139 | 33.9 |
| *missing* | *384* | 2.5 | 335 | 2.7 | 21 | 1.4 | 17 | 1.7 | 11 | 2.7 |
| **Number of living children** | | | | | | | | | | |
| 0 | 3818 | 24.5 | 3005 | 23.8 | 395 | 25.9 | 295 | 28.6 | 123 | 30.0 |
| 1 | 7871 | 50.5 | 6412 | 50.8 | 757 | 49.5 | 502 | 48.6 | 200 | 48.8 |
| 2 | 2477 | 15.9 | 2076 | 16.5 | 215 | 14.1 | 141 | 13.7 | 45 | 11.0 |
| 3 | 874 | 5.6 | 681 | 5.4 | 104 | 6.8 | 64 | 6.2 | 25 | 6.1 |
| missing | 551 | 3.5 | 446 | 3.5 | 57 | 3.7 | 31 | 3.0 | 17 | 4.2 |
| **Number of previous stillbirths/ pregnancy losses** | | | | | | | | | | |
| 0 | 14088 | 90.4 | 11451 | 90.7 | 1348 | 88.2 | 916 | 88.7 | 373 | 91.0 |
| 1 | 902.0 | 5.79 | 719 | 5.7 | 102 | 6.7 | 63 | 6.1 | 18 | 4.39 |
| 2 | 408.0 | 2.62 | 300 | 2.4 | 51 | 3.3 | 40 | 3.9 | 17 | 4.15 |
| 3 or more | 193.0 | 1.24 | 150 | 1.2 | 27 | 1.8 | 14 | 1.4 | 2 | 0.5 |
| **Previous experience with DHS surveys** | | | | | | | | | | |
| Yes | 11738 | 75.3 | 9607 | 76.1 | 1112 | 72.8 | 728 | 70.5 | 291 | 71.0 |
| No | 3469 | 22.3 | 2678 | 21.2 | 395 | 25.9 | 288 | 27.9 | 108 | 26.3 |
| *missing* | *384* | 2.5 | 335 | 2.7 | 21 | 1.4 | 17 | 1.7 | 11 | 2.7 |
| **Previous Experience with other surveys** | | | | | | | | | | |
| Yes | 9586 | 61.5 | 7729 | 61.2 | 921 | 60.3 | 667 | 64.6 | 269 | 65.6 |
| No | 5621 | 36.1 | 4556 | 36.1 | 586 | 38.4 | 349 | 33.8 | 130 | 31.7 |
| *missing* | 384 | 2.46 | 335 | 2.65 | 21 | 1.37 | 17 | 1.65 | 11 | 2.68 |

## Additional file 3.3: Distribution of missing year or month of pregnancy outcome by FBH+/FPH groups

|  | **FBH+** | | |  | **FPH** | | |  |
| --- | --- | --- | --- | --- | --- | --- | --- | --- |
|  | **Livebirths** | **Stillbirths^1^** | **Miscarriages^1^** | **p-value** | **Livebirths** | **Stillbirths** | **Miscarriages** | **p-value** |
| **Year of birth** | **n=98483** | **n=599** | **n=2981** | **p<0.0001** | **n=96816** | **n=1757** | **n=4300** | **p=0.266** |
| **Not imputed** | 98483 (100.0) | 599 (100.0) | 2833 (95.0) |  | 96725 (99.9) | 1757 (100.0) | 4298 (99.9) |  |
| **Imputed** | 0 (0.0) | 0 (0.0) | 148 (5.0) |  | 91 (0.1) | 0 (0.0) | 2 (0.1) |  |
| **Month of birth** | **n=98483** | **n=599** | **n=2981** | **p<0.0001** | **n=96816** | **n=1757** | **n=4300** | **p<0.0001** |
| **Not imputed** | 91389 (92.8) | 437 (73.0) | 1997 (66.7) |  | 90005 (93.0) | 1161 (66.1) | 2288 (53.2) |  |
| **Imputed** | 7094 (7.2) | 162(27.1) | 984 (33.0) |  | 6811 (7.0) | 596 (33.9) | 2012 (46.8) |  |

^1^ Due to survey design FBH+ includes only stillbirths and miscarriages occurring since 1^st^ Jan 2012.

**Imputation of missing dates**

We imputed dates for all pregnancy outcomes or children with missing or unknown dates of birth or death. We conducted a separate imputation for each missing/unknown day of birth/death, month of birth/death and year of birth/death using the following procedure:

***Year of birth/death*:**  For livebirths and post-neonatal survivors, we subtracted their current age from the year of interview; for deaths, we subtracted year of birth from age at death.

***Month of birth/death*:** We assigned a random number between 1 and 12. Each random number represented the months of year in a Gregorian calendar (i.e. 1 for January; 2 for February; 3 for March; 11 for November; and 12 for December etc.).

***Day of birth/death*:** We assigned a random number between 1 and 30 to represent the days of the month. During imputation we accounted for the differences in number of days in a calendar month and years (leap years or none leap years) i.e. for February in a leap year, we assigned a maximum possible random number-representing day of birth/death as 29. For calendar months with <30 or 30> days, a random numbers were assigned within the corresponding ceiling of the number of days.

## Additional file 3.4: Overview of response patterns to additional stillbirth classification questions

|  | Children surviving the neonatal period | | | | Neonatal Deaths | | | | Late gestation stillbirths | | | | Early gestation stillbirths | | | |
| --- | --- | --- | --- | --- | --- | --- | --- | --- | --- | --- | --- | --- | --- | --- | --- | --- |
|  | Total | Missing | Don’t know | % don’t know | Total | Missing | Don’t know | % don’t know | Total | Missing | Don’t know | % don’t know | Total | Missing | Don’t know | % don’t know |
| How many weeks pregnant were you when THIS BABY was born? (from Card or Recall) | 12,620 | 2 | 3,105 | (24.6%) | 1,528 | 1 | 581 | (38.0%) | 1,033 | 5 | 423 | (41.3%) | 410 | 2 | 174 | (42.4%) |
| Was THIS BABY born before expected? | 12,620 | 2 | 314 | (2.5%) | 1,528 | 1 | 56 | (3.7%) | 1,033 | 5 | 45 | (4.4%) | 410 | 3 | 28 | (6.8%) |
| How many weeks was THIS BABY born before the expected date of delivery? | 1,026 | 0 | 40 | (3.9%) | 394 | 0 | 86 | (21.9%) | 422 | 0 | 101 | (23.9%) | 338 |  | 140 | (41.4%) |
| When THIS BABY was born, was THIS BABY very large, larger than average, average, smaller than average, or very small? | 12,620 | 2 | 70 | (0.6%) | 1,528 | 1 | 88 | (5.8%) | 1,033 | 6 | 172 | (16.7%) | 410 | 3 | 157 | (38.3%) |
| Was THIS BABY weighed at birth? | 12,620 | 2 | 551 | (4.4%) | 1,528 | 1 | 195 | (12.8%) | 1,033 | 6 | 180 | (17.4%) | 410 | 3 | 63 | (15.4%) |
| How much did THIS BABY weigh? | 7,334 | 0 | 1,213 | (16.5%) | 701 | 0 | 179 | (25.5%) | 1634 | 0 | 21 | (12.8%) | 25 | 0 | 6 | (24.0%) |
| Did THIS BABY cry, move, or breathe at birth, even a little? | - |  |  |  | 1,528 | 1 | 15 | (1.0%) | 1,033 | 6 | 25 | (2.4%) | 410 | 3 | 26 | (6.3%) |
| If THIS BABY did not cry, move or breathe at birth, was he/she born dead?^1^ | - |  |  |  | 90 | 1 | 14 | (15.6%) | 1,006 | 6 | 25 | (2.5%) | 370 | 3 | 24 | (6.5%) |
| Did THIS BABY stop moving in the womb before labour pains started?^2^ | - |  |  |  | 47 | 0 | 5 | (10.6%) | 791 | 0 | 59 | (7.5%) | 251 |  | 28 | (11.2%) |
| Did a birth attendant listen for the baby's heart beat during labour with?^2^ | - |  |  |  | 47 | 0 | 6 | (12.8%) | 791 | 0 | 68 | (8.6%) | 251 |  | 18 | (7.2%) |
| Was the baby's heart beat present?^3^ | - |  |  |  | 24 | 0 | 11 | (45.8%) | 366 | 0 | 73 | (19.9%) | 93 |  | 17 | (18.3%) |
| Was the baby macerated; that is skin peeling or showing signs of decay?^2^ |  |  |  |  | 47 |  | 10 | (21.3%) | 791 |  | 151 | (19.1%) | 251 |  | 81 | (32.3%) |

Legend: Red=More than 20% missing, Yellow=Five to 20% missing, Green=less than 5% missing.

^1^Not asked to women responding that their baby cried, moved or breathed at birth.

^2^Only asked to women responding that their baby was born dead.

^3^Only asked to women responding that the birth attendant listened for the baby’s heartbeat.

^4^ Only asked for babies reported to be born dead and to have a congenital malformation.

## Additional file 3.5A: Distribution of reported gestational age in months by outcome (n=70,973)^1^

^1^5,458 pregnancy losses, 1,656 neonatal deaths, and 63,859 children surviving neonatal period.

## Additional file 3.5B: Distribution of reported gestational age in months by outcome (gestation ≥5 months) (n=66,793)^1^


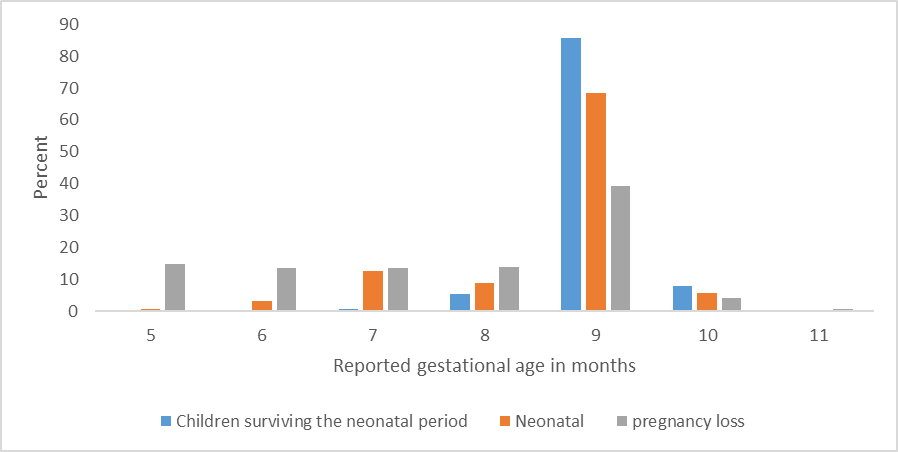


^1^1,510 stillbirths, 1,647 neonatal deaths, and 63,636 children surviving neonatal period.

## Additional file 3.6: Gestational age specific stillbirth, perinatal and early neonatal mortality rates, overall and site specific

**Gestational age specific stillbirth, perinatal and early neonatal mortality rates, five sites combined**

| Gestational Age From FBH+ or FPH | Total births  (stillbirth+ live birth) | Total live births | Gestation specific stillbirth mortality  /1000 Total Births | Gestation specific perinatal mortality /1000 Total Births | Gestation specific early neonatal mortality /1000  Live Births |
| --- | --- | --- | --- | --- | --- |
| 5 months | 242 | 19 | 921.5 | 971.1 | 631.6 |
| 6 months | 304 | 100 | 671.1 | 819.1 | 450.0 |
| 7 months | 920 | 713 | 225.0 | 403.3 | 230.0 |
| 8 months | 3,699 | 3,489 | 56.8 | 89.2 | 34.4 |
| 9 months | 56,247 | 55,653 | 10.6 | 27.0 | 16.6 |
| 10+ months | 5,381 | 5,309 | 13.4 | 29.5 | 16.4 |
| Overall | 66,793 | 65,283 | 22.6 | 42.8 | 20.7 |

Early neonatal mortality= neonatal death in the first 7 days of life after a livebirth (days 0-6). Perinatal mortality rate=(stillbirths+early neonatal deaths)/total births X1,000.

**Gestational-age specific stillbirth, perinatal and early neonatal mortality, Bandim**

| Gestational Age From FBH+ or FPH | Total births  (stillbirth+ live birth) | Total live births | Gestation specific stillbirth mortality  /1000 Total Births | Gestation specific perinatal mortality /1000 Total Births | Gestation specific early neonatal mortality /1000  Live Births |
| --- | --- | --- | --- | --- | --- |
| 5 months | 47 | 4 | 914.9 | 978.7 | 750.0 |
| 6 months | 58 | 14 | 758.6 | 810.3 | 214.3 |
| 7 months | 204 | 152 | 254.9 | 490.2 | 315.8 |
| 8 months | 87 | 36 | 586.2 | 724.1 | 333.3 |
| 9 months | 11273 | 11114 | 14.1 | 40.0 | 26.3 |
| 10+ months | 631 | 613 | 28.5 | 61.8 | 34.3 |
| Total | 12300 | 11933 | 29.8 | 60.7 | 31.8 |

Early neonatal mortality= neonatal death in the first 7 days of life after a livebirth (days 0-6). Perinatal mortality rate=(stillbirths+early neonatal deaths)/total births X1,000

**Gestational-age specific stillbirth, perinatal and early neonatal mortality, Dabat**

| Gestational Age From FBH+ or FPH | Total births  (stillbirth+ live birth) | Total live births | Gestation specific stillbirth mortality  /1000 Total Births | Overall Gestation specific perinatal mortality /1000 Total Births | Gestation specific early neonatal mortality /1000  Live Births |
| --- | --- | --- | --- | --- | --- |
| 5 months | 21 | 1 | 952.4 | 952.4 | 0.0 |
| 6 months | 24 | 4 | 833.3 | 833.3 | 0.0 |
| 7 months | 69 | 38 | 449.3 | 768.1 | 578.9 |
| 8 months | 48 | 26 | 458.3 | 812.5 | 653.8 |
| 9 months | 8237 | 8205 | 3.9 | 18.0 | 14.1 |
| 10+ months | 10 | 10 | 0.0 | 100.0 | 100.0 |
| Total | 8409 | 8284 | 14.9 | 33.4 | 18.8 |

Early neonatal mortality= neonatal death in the first 7 days of life after a livebirth (days 0-6). Perinatal mortality rate=(stillbirths+early neonatal deaths)/total births X1,000

| Gestational Age From FBH+ or FPH | Total births (stillbirth+ live birth) | Total live births | Gestation specific stillbirth mortality  /1000 Total Births | Overall Gestation specific perinatal mortality /1000 Total Births | Gestation specific early neonatal mortality /1000  Live Births |
| --- | --- | --- | --- | --- | --- |
| 5 months | 35 | 1 | 971.4 | 971.4 | - |
| 6 months | 55 | 26 | 527.3 | 727.3 | 423.1 |
| 7 months | 77 | 51 | 337.7 | 467.5 | 196.1 |
| 8 months | 197 | 179 | 91.4 | 177.7 | 95.0 |
| 9 months | 8028 | 7963 | 8.1 | 26.7 | 18.7 |
| 10+ months | 258 | 253 | 19.4 | 31.0 | 11.9 |
| Total | 8650 | 8473 | 20.5 | 42.4 | 22.4 |

**Gestational-age specific stillbirth, perinatal and early neonatal mortality, IgangaMayuge**

Early neonatal mortality= neonatal death in the first 7 days of life after a livebirth (days 0-6). Perinatal mortality rate=(stillbirths+early neonatal deaths)/total births X1,000

**Gestational-age specific stillbirth, perinatal and early neonatal mortality, Matlab**

| Gestational Age From FBH+ or FPH | Total births  (stillbirth + live birth) | Total live births | Gestation specific stillbirth mortality  /1000 Total Births | Overall Gestation specific perinatal mortality /1000 Total Births | Gestation specific early neonatal mortality /1000  Live Births |
| --- | --- | --- | --- | --- | --- |
| 5 months | 70 | 9 | 871.4 | 971.4 | 777.8 |
| 6 months | 97 | 42 | 567.0 | 845.4 | 642.9 |
| 7 months | 378 | 331 | 124.3 | 291.0 | 190.3 |
| 8 months | 3265 | 3189 | 23.3 | 41.3 | 18.5 |
| 9 months | 14184 | 14020 | 11.6 | 24.5 | 13.1 |
| 10+ months | 3342 | 3313 | 8.7 | 19.4 | 10.9 |
| Total | 21336 | 20904 | 20.2 | 37.9 | 18.0 |

Early neonatal mortality= neonatal death in the first 7 days of life after a livebirth (days 0-6). Perinatal mortality rate=(stillbirths+early neonatal deaths)/total births X1,000

| Gestational Age From FBH+ or FPH | Total births  (stillbirth + live birth) | Total live births | Gestation specific stillbirth mortality  /1000 Total Births | Overall Gestation specific perinatal mortality /1000 Total Births | Gestation specific early neonatal mortality /1000  Live Births |
| --- | --- | --- | --- | --- | --- |
| 5 months | 69 | 4 | 942.0 | 971.0 | 500.0 |
| 6 months | 70 | 14 | 800.0 | 857.1 | 285.7 |
| 7 months | 192 | 141 | 265.6 | 375.0 | 148.9 |
| 8 months | 102 | 59 | 421.6 | 568.6 | 254.2 |
| 9 months | 14525 | 14351 | 12.0 | 24.6 | 12.8 |
| 10+ months | 1140 | 1120 | 17.5 | 40.4 | 23.2 |
| Total | 16098 | 15689 | 25.4 | 41.0 | 16.0 |

**Gestational-age specific stillbirth, perinatal and early neonatal mortality, Kintampo**

Early neonatal mortality= neonatal death in the first 7 days of life after a livebirth (days 0-6). Perinatal mortality rate=(stillbirths+early neonatal deaths)/total births X1,000

## Additional file 3.7: Summary of reporting of gestational age in weeks for stillbirths

For the subset of births whose mothers were asked the additional questions (n=15,591), gestational age in weeks was more likely to be reported as unknown for stillbirths (41.6%), and neonatal deaths (38.0%) compared to children surviving the neonatal period (24.6%) (p<0.001). Gestational age in weeks were heaped on 28, 32, 36 and 38 weeks (Additional file 3.7A). Overall amongst those reporting gestational age in weeks the response was consistent with the reported gestational age in months for half of stillbirths (49.9%), compared to 39.5% of neonatal deaths and 30.2% of children surviving the neonatal period, with variation by site (Additional file 3.7B). Overall, for 42.5% of stillbirths, 44.7% of neonatal deaths and 58.9% of children surviving the neonatal period gestational age in weeks was exactly four times the reported gestational age in months. For 2.1% of stillbirths, 6.1% neonatal deaths and 3.0% of children surviving the neonatal period, reported gestational age in weeks was numerically identical to reported gestational age in months (Additional file 3.7C).

### Additional file 3.7A: Distribution of reported gestational age in weeks by outcome (n=15,591)


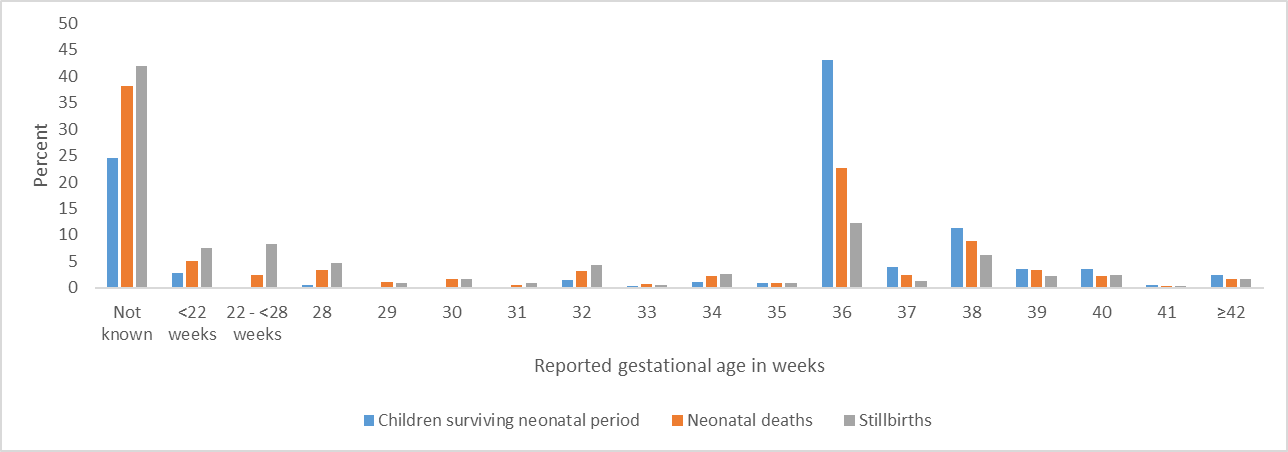


### Additional file 3.7B: Internal consistency of gestational age reported in weeks and months by site and by outcome^1^

|  | Overall | | Children surviving the neonatal period | | Neonatal deaths | | Stillbirths | |
| --- | --- | --- | --- | --- | --- | --- | --- | --- |
|  | Number reporting GA weeks | % consistent with GA months | Number reporting GA weeks | % consistent with GA months | Number reporting GA weeks | % consistent with GA months | Number reporting GA weeks | % consistent with GA months |
| Bandim | 109 | 27.5 | 80 | 32.5 | 16 | 18.8 | 13 | 7.7 |
| Dabat | 3044 | 4 | 2,761 | 2.9 | 181 | 5 | 102 | 33.3 |
| IgangaMayuge | 2132 | 24.2 | 1,780 | 23.3 | 194 | 22.7 | 158 | 36.1 |
| Matlab | 3586 | 70.8 | 2,738 | 71.5 | 433 | 67.7 | 415 | 69.6 |
| Kintampo | 2427 | 18.9 | 2,154 | 18.4 | 122 | 20.5 | 151 | 25.2 |
| Overall | 11298 | 32.5 | 9,513 | 30.2 | 946 | 39.5 | 839 | 49.9 |

^1^ Internal consistency defined as responses to gestational age in weeks questions being equal to ((gestational age in months)X4.33)+/- 2.

### Additional file 3.7C: Distribution of reported gestational age in weeks for pregnancies lasting <28 weeks

|  | Livebirths surviving neonatal period | Neonatal deaths | Stilbirths^1^ |
| --- | --- | --- | --- |
| How long did your pregnancy last? | | | |
| Total <28 weeks | 364 | 112 | 228 |
| 22 - <28 weeks | 7 | 36 | 119 |
| 18 - <22 | 15 | 4 | 69 |
| <18 | 342 | 72 | 40 |

^1^ Defined as a pregnancy loss at 5 or more months based on responses in the FBH+ or FPH.

Gestational ages at birth of less than 22 weeks for infants surviving the neonatal period and less than 18 weeks for neonatal deaths are considered to be biologically implausible (highlighted in orange in the table above). In contrast, for pregnancy losses any gestational age may be plausible.

|  | All  outcomes | Livebirths surviving neonatal period | Neonatal deaths | Stilbirths^1^ |
| --- | --- | --- | --- | --- |
| Total | 15,591 | 12,620 | 1,528 | 1,443 |
| Reporting GA in weeks | 11,297 | 9,513 | 946 | 838 |
| Total <18 weeks | 454 | 342 | 72 | 40 |
| Reported GA weeks= reported GA months | | | | |
| Total | 358 | 282 | 58 | 18 |
| Breakdown of those with GA weeks=GA months by reported GA | | | | |
| 5 | 2 | 0 | 0 | 2 |
| 6 | 1 | 0 | 0 | 1 |
| 7 | 19 | 3 | 14 | 2 |
| 8 | 22 | 13 | 7 | 2 |
| 9 | 177 | 148 | 22 | 7 |
| 10 | 137 | 118 | 15 | 4 |

Of the 454 livebirths and pregnancy losses at 5 or more months where the mother responded that the pregnancy lasted fewer than 18 weeks, the reported gestational age in weeks was numerically identical to the reported gestational age in months in 358 (78.9%). This is likely to have occurred due to confusion or error by either the mother or the interviewer [1].

## Additional file 3.8: Birthweight distribution by outcome

**Birthweight distribution for children surviving the neonatal period n=6,121. (53.3% heaped on multiples of 500g)**


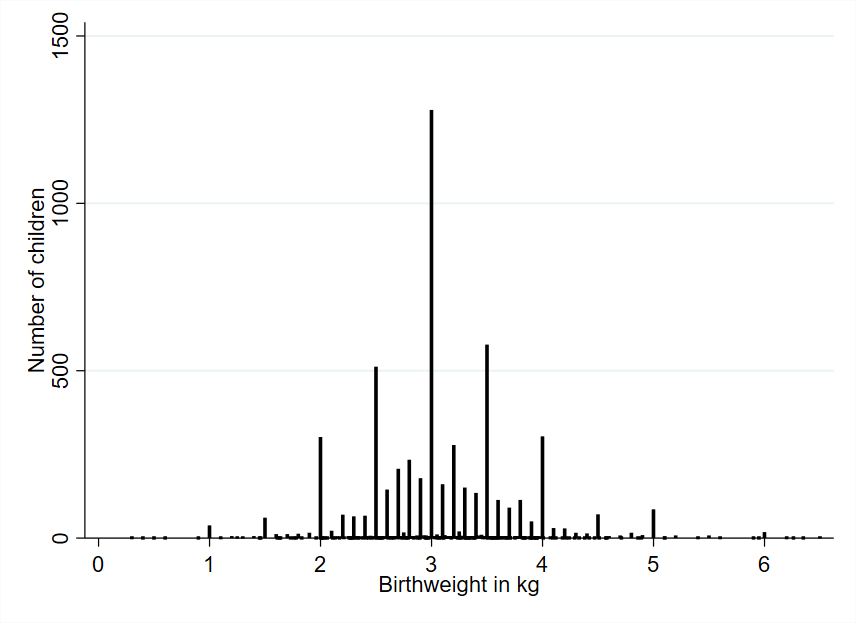


**Birthweight distribution for neonatal deaths n=522. (57.9% heaped on multiples of 500g)**


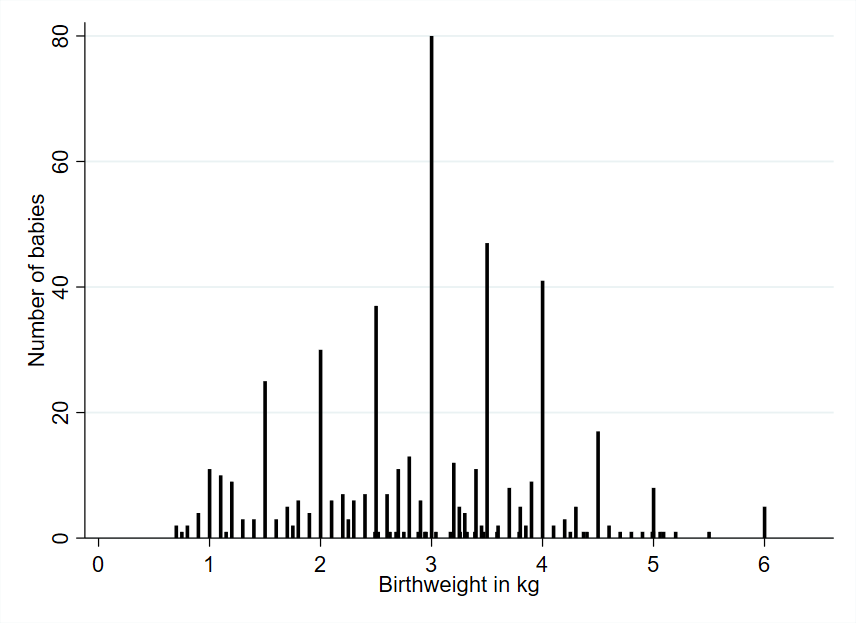


**Birthweight distribution for late gestation stillbirths n=143. (61.5% heaped on multiples of 500g)**

**
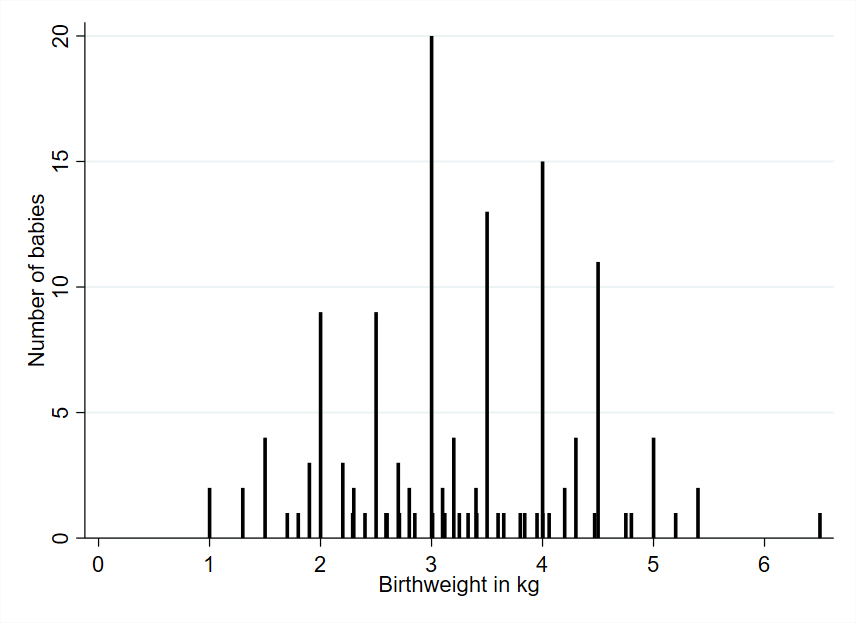
**

**Birthweight distribution for early gestation stillbirths n=19. (47.7% heaped on multiples of 500g)**

**
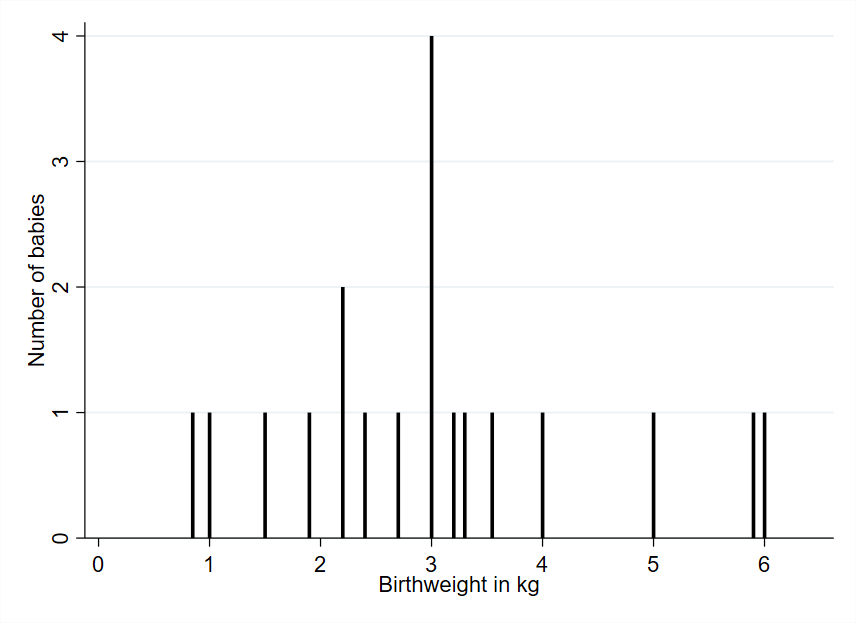
**

## Additional file 3.9: Heaping indices for birthweight, by outcome (n=6,749)

|  | **Children surviving the**  **neonatal period** | **Neonatal deaths** | **Late gestation**  **Stillbirths** | **Early gestation Stillbirths** |
| --- | --- | --- | --- | --- |
| Total with valid weight | 6075 | 513 | 142 | 19 |
| 1,000g | 4.1 | 0.4 | NA | 1.0 |
| 1,500g | 1.5 | 1.8 | 1.3 | NA |
| 2,000g | 2.2 | 1.4 | 1.3 | 0.0 |
| 2,500g | 0.9 | 1.0 | 0.9 | 0.0 |
| 3,000g | 1.3 | 2.2 | 1.8 | 4.0 |
| 3,500g | 0.9 | 1.5 | 2.0 | 0.0 |
| 4,000g | 1.1 | 1.8 | 2.5 | NA |
| 4,500g | 1.3 | 1.9 | 2.2 | NA |
| 5,000g | 2.3 | 1.1 | 2.0 | NA |

Heaping Indices were calculated as: Number of babies with a given birthweight / number of babies with a birthweight within 249g above or below the given birthweight

e.g. Number exactly on 1,500g / (Number (1,251g to 1,499g)+ Number(1,501g to 1,749g))

NA=no babies with that outcome reporting the given birthweight

## Additional file 3.10: Comparison of heaping on multiples of 500g by card and recall by outcome

**Children surviving the neonatal period (Card n=2,525 (32.9% heaped) Recall n=3,596 (67.6% heaped)**

**
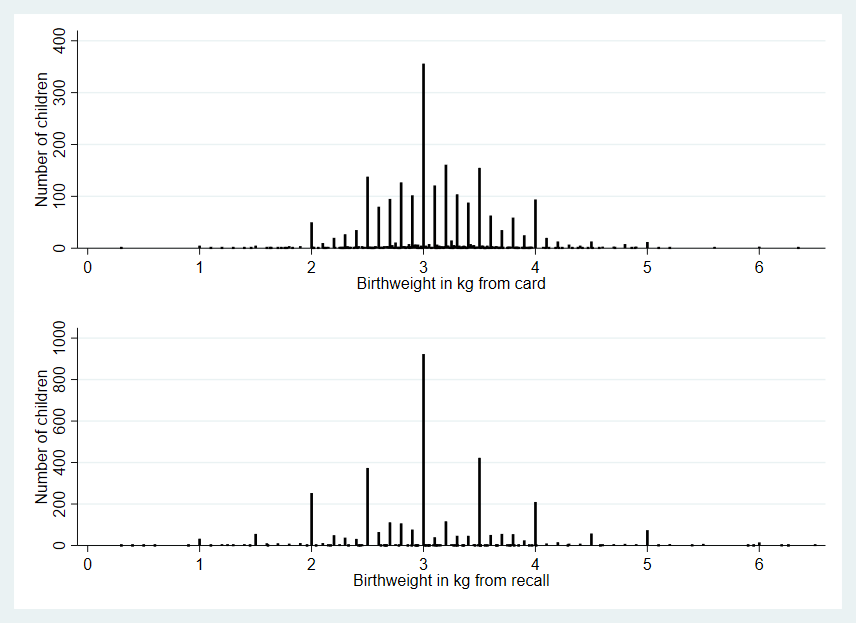
**

**Neonatal deaths (Card n=71 (40.9% heaped). Recall n=4521 (60.5% heaped))**

**
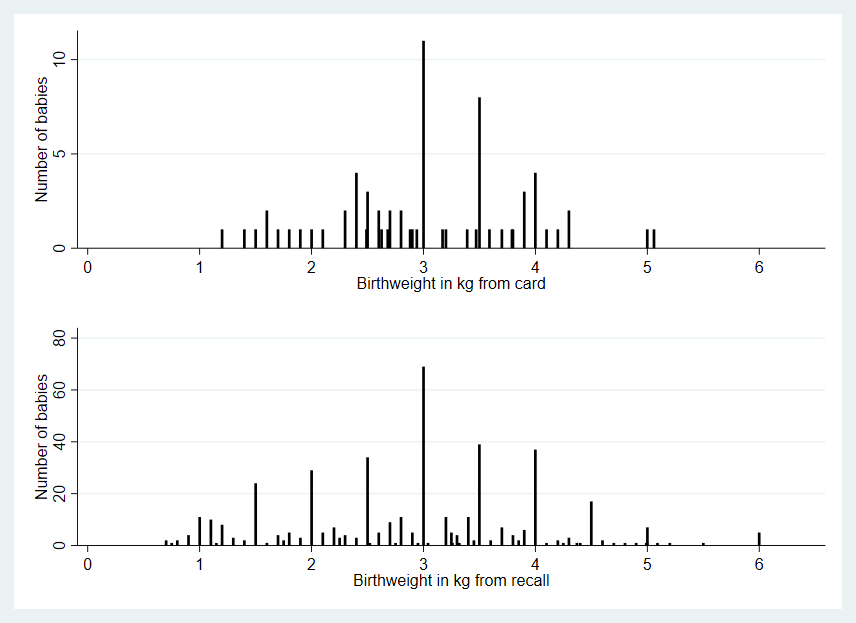
**

**Late gestation stillbirths (Card=22 (31.8% heaped). Recall n=121 (66.9% heaped))**


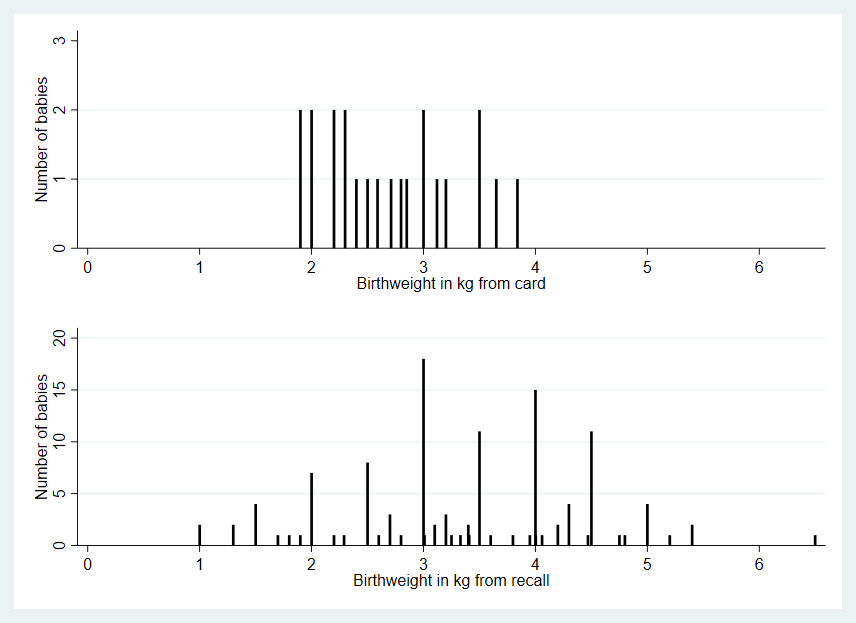


**Early gestation stillbirths (Card n=10 (40% heaped). Recall n=9 (55.6% heaped))**

**
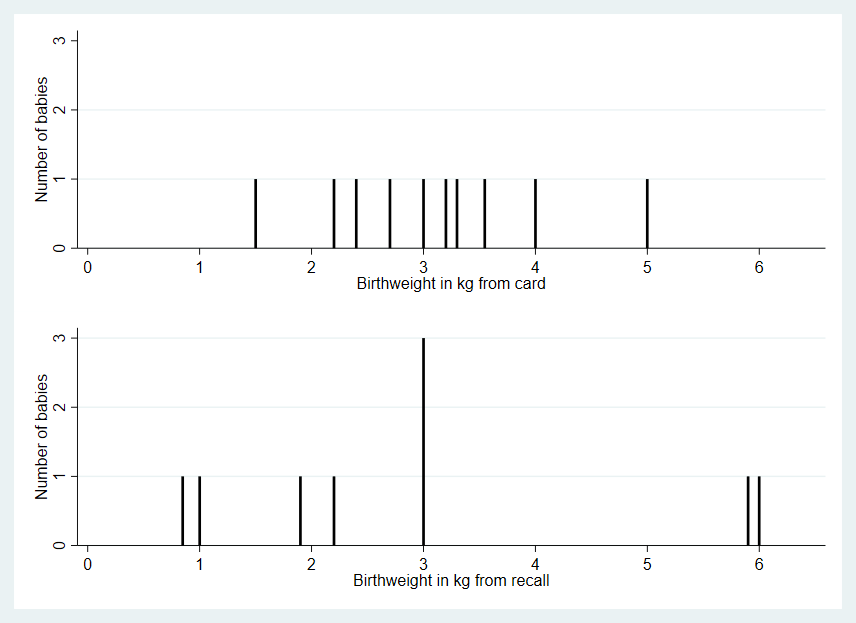
**

## Additional file 3.11: Reported intrapartum monitoring for late gestation stillbirths, by site (n=846)

|  |  | All births | | Facility births | | Home births | |
| --- | --- | --- | --- | --- | --- | --- | --- |
| Overall | Yes | 366 | 43.3 | 354 | 55.6 | 12 | 5.7 |
|  | doppler | 165 | 19.5 | 157 | 24.6 | 8 | 3.8 |
|  | stethoscope | 201 | 23.8 | 197 | 30.9 | 4 | 1.9 |
|  | No | 401 | 47.4 | 207 | 32.5 | 194 | 92.8 |
|  | Don't Know | 79 | 9.3 | 76 | 11.9 | 3 | 1.4 |
| Bandim | Yes | 69 | 43.4 | 64 | 46.7 | 5 | 22.7 |
|  | doppler | 30 | 18.9 | 27 | 19.7 | 3 | 13.6 |
|  | stethoscope | 39 | 24.5 | 37 | 27.0 | 2 | 9.1 |
|  | No | 56 | 35.2 | 40 | 29.2 | 16 | 72.7 |
|  | Don't Know | 34 | 21.4 | 33 | 24.1 | 1 | 4.5 |
| Dabat | Yes | 18 | 25.4 | 17 | 37.0 | 1 | 4.0 |
|  | doppler | 10 | 14.1 | 10 | 21.7 | 0 | 0.0 |
|  | stethoscope | 8 | 11.3 | 7 | 15.2 | 1 | 4.0 |
|  | No | 40 | 56.3 | 16 | 34.8 | 24 | 96.0 |
|  | Don't Know | 13 | 18.3 | 13 | 28.3 | 0 | 0.0 |
| Iganga | Yes | 49 | 53.3 | 49 | 62.0 | 0 | 0.0 |
|  | doppler | 33 | 35.9 | 33 | 41.8 | 0 | 0.0 |
|  | stethoscope | 16 | 17.4 | 16 | 20.3 | 0 | 0.0 |
|  | No | 30 | 32.6 | 17 | 21.5 | 13 | 100.0 |
|  | Don't Know | 13 | 14.1 | 13 | 16.5 | 0 | 0.0 |
| Matlab | Yes | 93 | 32.4 | 91 | 47.9 | 2 | 2.1 |
|  | doppler | 36 | 12.5 | 35 | 18.4 | 1 | 1.0 |
|  | stethoscope | 57 | 19.9 | 56 | 29.5 | 1 | 1.0 |
|  | No | 189 | 65.9 | 94 | 49.5 | 95 | 97.9 |
|  | Don't Know | 5 | 1.7 | 5 | 2.6 | 0 | 0.0 |
| Kintampo | Yes | 137 | 57.8 | 133 | 71.9 | 4 | 7.7 |
|  | doppler | 56 | 23.6 | 52 | 28.1 | 4 | 7.7 |
|  | stethoscope | 81 | 34.2 | 81 | 43.8 | 0 | 0.0 |
|  | No | 86 | 36.3 | 40 | 21.6 | 46 | 88.5 |
|  | Don't Know | 14 | 5.9 | 12 | 6.5 | 2 | 3.8 |

Data available for 846 out of 1033 total late fetal deaths

## Additional file 3.12: Individual-level consistency between intrapartum stillbirth classification, by site

|  | Fetal movement and fetal heart sounds | | | Fetal movement and skin appearance | | | Fetal heart sounds and skin appearance | | |
| --- | --- | --- | --- | --- | --- | --- | --- | --- | --- |
|  | Consistent IPSB classification | Non consistent IPSB classification | % of women reporting consistent responses | Consistent IPSB classification | Non consistent IPSB classification | % of women reporting consistent responses | Consistent IPSB classification | Non consistent IPSB classification | % of women reporting consistent responses |
| Overall | 47 | 319 | 14.7 | 220 | 626 | 35.1 | 59 | 307 | 19.2 |
| Bandim | 4 | 65 | 6.2 | 23 | 136 | 16.9 | 3 | 66 | 4.5 |
| Dabat | 3 | 15 | 20.0 | 24 | 47 | 51.1 | 2 | 15 | 13.3 |
| IgangaMayuge | 11 | 38 | 28.9 | 26 | 66 | 39.4 | 13 | 36 | 36.1 |
| Matlab | 13 | 80 | 16.3 | 88 | 199 | 44.2 | 21 | 72 | 29.2 |
| Kintampo | 16 | 121 | 13.2 | 59 | 178 | 33.1 | 19 | 118 | 16.1 |

Information to categorise based on fetal heart sounds was limited to the women reporting that the attendant listened for fetal heart sounds (n=366).

## Additional file 3.13: Responses to congenital malformation questions for stillbirths in 5 years prior to survey n=1443^1^

| Number reported presence of congenital malformations (% of all stillbirths) | Yes (single) | 39  (2.7%) |
| --- | --- | --- |
|  | Yes (multiple) | 6  (0.4%) |
|  | No | 839 (58.1%) |
|  | Don’t know | 246 (17.0%) |
|  | Missing | 308 (21.3%) |
| Types of single malformation (n=39) | Don’t know | 3 |
|  | Spinal defects | 8 |
|  | Hydro-/Macrocephaly | 1 |
|  | Microcephaly | 4 |
|  | Cleft lip/ palate | 0 |
|  | Abdominal wall defect | 2 |
|  | Other | 21 |
| Types of multiple malformations (n=6) | Microcephaly and 'other' | 2 |
|  | Hydro-/Macrocephaly and other | 1 |
|  | Spinal defects and multiple other malformations | 2 |
|  | Cleft lip/ palate and abdominal wall defect | 1 |

^1^ Includes both early and late gestation stillbirths.

## Additional file 3.14: Reporting of child’s sex by site and outcome (FPH group only. n=631)

| Study site | Early gestation stillbirths^1^  (%) | | | | Late gestation stillbirths  (%) | | | | Neonatal deaths  (%) | | | Children surviving neonatal period | | |
| --- | --- | --- | --- | --- | --- | --- | --- | --- | --- | --- | --- | --- | --- | --- |
|  | Total asked | Male | Female | Don’t know | Total  asked | Male | Female | Don’t know | Total  asked | Male | Female | Total  asked | Male | Female |
| Overall | 91 | 47.3 | 24.2 | 28.6 | 540 | 55.2 | 38.7 | 6.1 | 1,528 | 62.2 | 37.8 | 12,620 | 50.0 | 50.0 |
| Bandim | 20 | 60.0 | 15.0 | 25.0 | 141 | 56.0 | 39.0 | 5.0 | 404 | 61.6 | 38.4 | 1290 | 50.4 | 49.6 |
| Dabat | 12 | 25.0 | 8.3 | 66.7 | 39 | 64.1 | 23.1 | 12.8 | 191 | 63.4 | 36.6 | 3,025 | 48.9 | 51.1 |
| IgangaMayuge | 11 | 45.5 | 36.4 | 18.2 | 76 | 50.0 | 42.1 | 7.9 | 199 | 55.8 | 44.2 | 1,811 | 49.7 | 50.3 |
| Matlab | 29 | 58.6 | 31.0 | 10.3 | 135 | 55.6 | 44.4 | 0.0 | 437 | 63.8 | 36.2 | 2,745 | 50.7 | 49.3 |
| Kintampo | 19 | 31.6 | 26.3 | 42.1 | 149 | 54.4 | 35.6 | 10.1 | 297 | 64.0 | 36.0 | 3,749 | 50.3 | 49.7 |

Sex was only asked to women with stillbirths in the FPH group .

^1^ Sex of stillbirth was not asked for losses at 5 months pregnancy duration.

## Additional file 3.15: Barriers and enablers to birthweight measurement in surveys for stillbirths by site

|  | Bandim | Dabat | IgangaMayuge | Matlab | Kintampo |  |
| --- | --- | --- | --- | --- | --- | --- |
| **Barriers to birthweight measurement** | | | | | | |
| Child deceased or stillbirth | ✓  No perceived benefit in weighing a dead child (especially if at home) |  | ✓  No perceived benefit in weighing stillborn children | ✓  All perceived no benefit in weighing stillborn children.  Some mothers reported that when anyone gave birth to a stillborn baby at facility the nurses showed an attitude of very negligence instead of measuring the weight of the baby. They perceive that this was because stillborn babies might bring down the reputation of the hospital. | ✓  No perceived benefit in weighing stillborn children or those who die shortly after birth |  |
| **Barriers to reporting birthweight** | | | | | | |
| Mother not informed of the weight | ✓  Baby separated from mother soon after birth e.g. very sick or stillborn. Mother perceived that even if the baby was weighed they may not be told what the weight was. |  |  |  |  |  |
| **Enablers to being weighed** | | | | | | |
| Perceived value of birthweight for stillbirths | ×  Reported none |  | ✓  Can help health workers know cause |  | ✓  Can help plan you know what to eat in a subsequent pregnancy to avoid another stillbirth  Can help health workers know cause |  |
